# Targeting DNA Homologous Repair Proficiency With Concomitant Topoisomerase II and c-Abl Inhibition

**DOI:** 10.3389/fonc.2021.733700

**Published:** 2021-09-20

**Authors:** Arafat Siddiqui, Manuela Tumiati, Alia Joko, Jouko Sandholm, Pia Roering, Sofia Aakko, Reetta Vainionpää, Katja Kaipio, Kaisa Huhtinen, Liisa Kauppi, Johanna Tuomela, Sakari Hietanen

**Affiliations:** ^1^Institute of Biomedicine, University of Turku, Turku, Finland; ^2^ONCOSYS, Research Programs Unit, University of Helsinki, Helsinki, Finland; ^3^Department of Biology, Åbo Akademi University, Turku, Finland; ^4^Turku Bioscience Centre, University of Turku and Åbo Akademi University, Turku, Finland; ^5^Laboratory of Genetics, HUS Diagnostic Center, Helsinki University Hospital, Helsinki, Finland; ^6^Turku University Hospital, FICAN West Cancer Centre, Turku, Finland

**Keywords:** DNA repair, cell cycle arrest, c-Abl, imatinib, mitoxantrone

## Abstract

Critical DNA repair pathways become deranged during cancer development. This vulnerability may be exploited with DNA-targeting chemotherapy. Topoisomerase II inhibitors induce double-strand breaks which, if not repaired, are detrimental to the cell. This repair process requires high-fidelity functional homologous recombination (HR) or error-prone non-homologous end joining (NHEJ). If either of these pathways is defective, a compensatory pathway may rescue the cells and induce treatment resistance. Consistently, HR proficiency, either inherent or acquired during the course of the disease, enables tumor cells competent to repair the DNA damage, which is a major problem for chemotherapy in general. In this context, c-Abl is a protein tyrosine kinase that is involved in DNA damage-induced stress. We used a low-dose topoisomerase II inhibitor mitoxantrone to induce DNA damage which caused a transient cell cycle delay but allowed eventual passage through this checkpoint in most cells. We show that the percentage of HR and NHEJ efficient HeLa cells decreased more than 50% by combining c-Abl inhibitor imatinib with mitoxantrone. This inhibition of DNA repair caused more than 87% of cells in G2/M arrest and a significant increase in apoptosis. To validate the effect of the combination treatment, we tested it on commercial and patient-derived cell lines in high-grade serous ovarian cancer (HGSOC), where chemotherapy resistance correlates with HR proficiency and is a major clinical problem. Results obtained with HR-proficient and deficient HGSOC cell lines show a 50–85% increase of sensitivity by the combination treatment. Our data raise the possibility of successful targeting of treatment-resistant HR-proficient cancers.

**Graphical Abstract:**
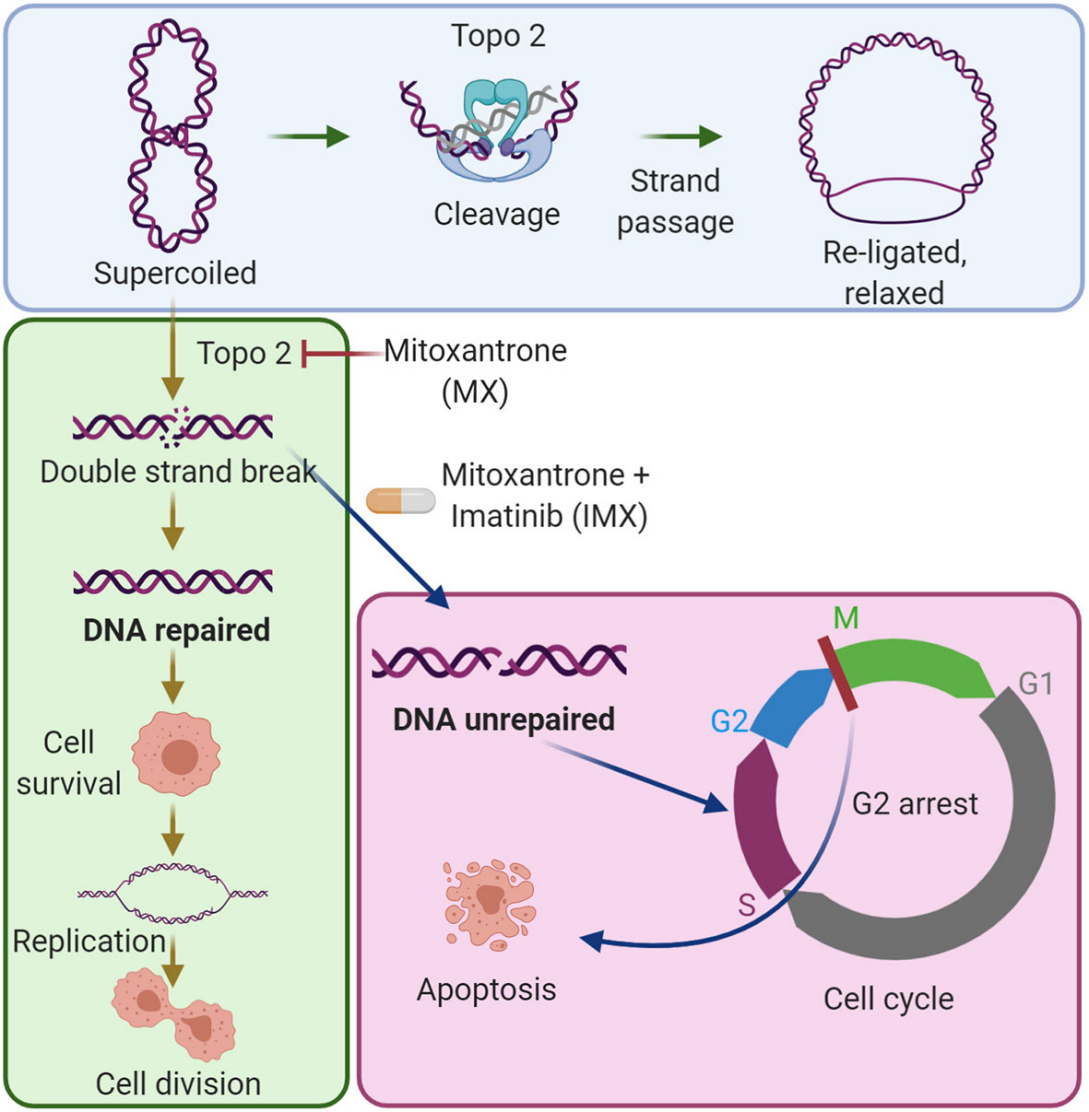
The combination of imatinib with low-dose mitoxantrone inhibits DNA repair in cancer cells. The blue area shows the established mechanism of the type II topoisomerase (Topo 2) enzyme in DNA synthesis. The green area shows that low-dose mitoxantrone inhibits Topo 2 and causes DNA double-strand breaks, which can be repaired by cancer cells, and by this, the cells can survive the damage. The magenta area shows that the combination of imatinib with mitoxantrone prevents DNA repair, causes G2/M cell cycle arrest, and induces apoptosis.

## Introduction

DNA damage repair (DDR) plays a critical role in the maintenance of genomic stability. An appropriate response to genotoxic drugs is required for cancer cell survival. Targeting this response has become an active area of research in the field of developing new cancer therapies. DNA damage alerts surveillance networks that stall cell cycle, allowing time for DNA repair. High in the hierarchy of these pathways lie the activities of ATM and ATR, which belong to the phosphatidylinositol 3-kinase-related protein kinase family (1–3). DNA repair involves mainly six pathways, including mismatch repair, homologous recombination repair, non-homologous end joining, trans-lesion DNA synthesis, base excision repair, and nucleotide excision (4). The most common pathways to repair double-stranded breaks (DSBs) are non-homologous end joining (NHEJ) and homologous recombination (HR). NHEJ is error-prone and active throughout the cell cycle, whereas HR is a high-fidelity repair mechanism limited to the S and G2 phases of the cell cycle after genotoxic stress (5).

Induction of genotoxic stress response is one of the hallmarks of chemotherapy. However, resistance to genotoxic chemotherapies is a problem in HR-proficient (HRP) cancers. HRP cancers can repair the damage, resulting in treatment resistance (6). This is a very common case in high-grade serous ovarian cancer (HGSOC), which is the most lethal gynecological cancer and is mostly treated by surgery together with platinum-based therapy. However, HGSOC becomes resistant to platinum-based therapy and most patients relapse within 2 years (7). In contrast, HR-deficient (HRD) cancers are usually sensitive to treatment. Recent pivotal clinical studies have shown that Poly (ADP-ribose) polymerase (PARP) inhibitors have dramatically improved in the prognosis of HRD cancers (8–10). However, HRD cancers can also regain partial HR function, making them resistant to these inhibitors (11, 12). This development closely correlates with HR-proficiency and makes HGSOC a difficult-to-treat disease. There is an unmet clinical need for treatment that can abrogate HR-proficiency.

Type II topoisomerases are essential for resolving DNA tangles and supercoils during replication. They cut both daughter strands while simultaneously passing another duplex DNA through the break, and then rejoin the broken strands (8). Failure in strand separation leads to cell death, which is the mechanistic principle behind the use of topoisomerase II inhibitors as a chemotherapeutic. Topoisomerase II inhibitors have been successfully used in cancer therapy for over 30 years but they display considerable tissue toxicity. Mitoxantrone (MX) is a topoisomerase II inhibitor that leads to accumulation of DNA crosslinks as well as single- and double-strand breaks. In DNA damage repair by HR, the c-Abl proto-oncoprotein has a direct role, where it phosphorylates RAD51 at Tyr-315 (13). Once in the nucleus, the phosphorylated RAD51 co-localizes with BRCA1 at DSB sites. Activation of c-Abl at the G1-S transition ensures DNA repair capability when replication progresses into S phase (14, 15). It has been reported that c-Abl activity increases in three cases. First, c-Abl is activated in an ATM-dependent manner as response to DNA damage following genotoxic stress (16). Second, in chronic myeloid leukemia, c-Abl is constitutively active due to its fusion with breakpoint cluster region protein (BCR), promoting DNA repair (17, 18). Third, overexpression of wild type c-Abl may occur in solid tumors in non-stressed conditions, such as in aggressive types of breast cancer and non-small cell lung cancer (19).

Here, we show that combining low dose topoisomerase II inhibitor mitoxantrone with c-Abl inhibitor imatinib (IM) effectively impairs DNA repair in both HR-deficient and -proficient cells. We validated the findings in HGSOC cells, where HR proficiency is a key problem in both chemotherapy resistance and PARP inhibitor resistance.

## Materials and Methods

### Cell Lines and Cytotoxic Drugs

HeLa cell line was used as a 2D cancer cell model for microarray analysis and DNA-repair assay. FUCCI-HeLa was used for functional validation of G2/M arrest. BRCA1-wt MDA-MB-231 and BRCA1-mutant HCC-1937 cell lines were used to perform other *in vitro* assays (**Supplementary Table S1**).

We used total 13 patient-derived and conventional HGSOC cell lines to validate cytotoxicity of MX *versus* IMX. Patient-derived cell lines include M022p, M022i, M048i, H002, and OC002. Commercial cell lines include COV318, CaOV3, OVCAR3, OVCAR4, OVCAR5, COV362, Kuramochi, and OVCAR8 (**Supplementary Tables S1, S2**). Patient-derived cell lines were developed from tissue and ascites specimen, which were collected from consented high-grade serous ovarian cancer (HGSOC) patients at Department of obstetrics & gynaecology, Turku University Hospital (TYKS) as described previously (20). Tissue samples and clinical information were collected from four patients diagnosed with stage III or IV as described earlier. Treatment-naïve ascites was collected during diagnostic laparoscopy (cell line OC002). Patients considered primarily inoperable received three cycles of neoadjuvant chemotherapy (NACT), and new samples were taken during interval debulking surgery (cell lines M022i, M048i, and H002i). Ascites samples (cell lines OC002, M022i, and H002i) were gradient centrifuged with Histopaque-1077 concentrate the cancer cells and plated. Omentum tumor sample (cell line M048i) was minced into approximately 1 mm^3^ pieces and plated on six-well plates. The stromal and immune cells were grown out by passaging approximately five times. SBS3 is considered as strong predictor of defective HR-based repair (21). Functional HR-score was also performed for all cell lines (22). All cell lines were grown in either DMEM or RPMI-1640 with additional supplements under standard cell culture condition (**Supplementary Table S3**). DMSO (0.1%), mitoxantrone, and imatinib mesylate were used as treatments (**Supplementary Table S4**).

### Microarray Analysis

Total RNA from triplicate treatments of 5 µM IM, 0.8 µM MX, or their combinations for 30 h was extracted using Trizol and further purified with RNeasy RNA isolation kit according to manufacturer’s instructions (**Supplementary Table S4**). The quality of RNA was controlled before hybridization, and one low quality sample (MX) was excluded from the analysis. The analysis was done on an Illumina HumanRafSeq-8v2 chip containing 22184 transcripts. Quantile normalisation method was applied after hybridization to remove non-biological variation. Data similarity between replicates was confirmed with Pearson coefficient metrics and Principal Component Analysis. Treatment group comparisons were performed with R-language limma package.

### Pathway Enrichment Analysis

Gene Set Enrichment Analysis (GSEA v. 4.0.3) ref) was performed using GOPB_AllPathways_no_GO_ie as reference gene set:

(http://download.baderlab.org/EM_Genesets/current_release/Human/symbol/)

A total of 1000 gene permutations (term size from 15 to 300) were used to generate a null distribution of enrichment score (ES), and then each pathway will attain normalization ES (NES). FDR Q-value (false discovery rate) <0.1 and p value < 0.01 were considered significant. Volcano plots of each full data set were generated with Galaxy server (23). The functional network was constructed with the GSEA data fed into Cytoscape v. 3.8.0, where both up- and downregulated pathways can be visualized simultaneously (24). Most significant pathway nodes were filtered with Diffusion plugin.

### Cell Viability and IC_50_ Measurement

Cells (2500–5000 cells per well) were grown in 96-well plates for 24 h prior to drug treatment. IC_50_ values of MX with and without IM were determined using CCK-8 kit (**Supplementary Table S4**). The MX dose started from 4000 nM and was followed by 50% serial dilutions to lower doses until 0.0 nM of MX. In all groups, we used constant 5 µM IM. DMSO (0.1%) was used as a vehicle control. Relative absorbance at 450 nm was measured after 72 h treatment. The IC_50_ value was obtained by non-linear regression analysis using Graph Pad Prism 8.4.2 (GraphPad Software, San Diego, USA). Additional cell viability experiments were performed to confirm the IC_50_ values. Cells were treated with vehicle, IM, MX, and IM+MX (IMX) for 72 h followed by CCK8-assay and optical density measurement, as described earlier. Based on these measurements, working drug concentrations were determined for individual cell lines, and these were used throughout the study, as follows: 34 nM MX and 5 µM IM for HeLa and FUCCI-HeLa, 103.5 nM MX and 5 µM IM for MDA-MB-231, and 9 nM MX and 5 µM IM for HCC-1937.

### Live Cell Imaging

Vehicle or indicated concentrations of drugs were added to cells (2500–5000 cells per well in a 96-well plate). Cells were imaged at 5 min to 1 h intervals for 5–10 days using IncuCyte S3 imaging system (Essen Bioscience, Michigan, USA) with a 20x objective. IncuCyte 2019B Rev2 software (Essen BioScience Michigan, USA) was used to calculate mean confluences (three parallel wells per treatment) from phase contrast images. For FUCCI-HeLa cells, fluorescence channels were used. Representative wells were selected for time-lapse movies, which were built with ImageJ ver 1.47d (25).

### Flow Cytometry

All flow cytometry experiments were performed on a BD LSRFortessa™ flow cytometer (BD Biosciences). Flowing Software 2.5.1 (Mr Perttu Terho, Turku Bioscience Centre, Turku, Finland) and FlowJo ver 10 (Tree Star Inc) were used for analysis.

### Nuclear Fractionation for RAD51 Measurement

HeLa cells (200,000 cells per well were seeded on six-well plates and incubated overnight) were treated with 0.1% DMSO, 5 µM IM, 34 nM MX, and IMX for 24 h. Then, cells were subjected to nuclear fractionation followed by staining with rabbit α-RAD51 primary antibody and donkey anti-rabbit IgG-AlexaFluor 488 secondary antibody (**Supplementary Tables S4, S5**). Samples were analyzed by flow cytometry. In addition to nuclear fractions, whole cell samples were analyzed to validate the fractionation process where whole cell population had different position than nuclear population in FSC/SSC plot.

### Cell Cycle Analysis With EdU and FUCCI-HeLa Cells

Assay kit was used to assess the treatment effect on cell cycle of HeLa, MDA-MB-231, and HCC-1937 cells. Cells (200,000) per well were seeded on six-well plates followed by overnight incubation. Cells were treated for 24–72 h with 0.1% DMSO, 5 µM IM, and individual doses of MX and IMX per cell lines. The samples were analyzed by flow cytometry.

FUCCI-HeLa cells were seeded at 20,000 per well in 24-well plates. After 24 h of treatment with vehicle, 5 µM IM, 34 nM MX, or 5 µM IM + 34 nM MX (IMX), cell pellets were collected and fixed with fixation buffer followed by flow cytometry analysis (**Supplementary Table S4**).

### RNA Interference and Western Blot Analysis

FlexiTube siRNA BRCA1 (5 nmol) and 0.3 ml Lipofectamine were used to silence BRCA1 in MDA-MB-231 and FUCCI-HeLa cell lines. For Western blot, cells were lysed in RIPA buffer supplemented with protease inhibitor. Protein concentrations were determined with BCA method. 30 µg protein was loaded on SDS-PAGE gels and transferred to nitrocellulose membranes. The membranes were incubated with BRCA1 antibody (**Supplementary Tables S4, S5**). Detection was done with Odyssey Imager (LI-COR Biosciences). After image acquisition, the obtained images were analyzed using Image Studio Lite (Version 5.0).

### Immunocytochemistry (ICC)

Cells were seeded on coverslips and treated with vehicle or indicated doses of MX, IM, and IMX. After 8 h or 24 h, cells were fixed with 2% buffered PFA for 10 min, permeabilized for 20 min in 0.2% Triton X-100/PBS, and washed thrice with 1% BSA/0.05% Tween/PBS. To reduce unspecific signal, cells were blocked in 1% BSA/10% Normal Donkey Serum/PBS for 30 min and then incubated overnight at +4°C with the following primary antibodies: α-γH2Ax, α-RAD51, α-cleaved Caspase-3, and actin. Cells were subsequently washed thrice with 1% BSA/0.05% Tween/PBS and incubated for 1 h at room temperature with fluorescently labeled secondary antibodies. Nuclei were counterstained with Hoechst 33342. Stained coverslips were mounted with ProlongGold. Images were acquired with a Nikon 90i Eclipse microscope (10x) and analyzed with the NIS Elements software. MDA-MB-231 (BRCA1-wt and silenced) cells were stained with α-γH2Ax and RAD51 antibodies. HeLa and HCC-1937 cells were stained with α-γH2Ax and α-cleaved Caspase-3 antibodies (**Supplementary Tables S4, S5**).

### DNA Double Strand Break Repair Assay

HeLa cells with pDRGFP (HR plasmid construct) and pimEJ5GFP (NHEJ plasmid construct) were transfected with pCBASceI, a I-SceI endonuclease expression vector with a mammalian promoter to introduce a DSB at a genomic I-SceI site of DNA repair plasmid constructs. Cells were grown for 4 days followed by flow cytometry analysis with Alexa Fluor 488 and mCherry channel. mCherry was used as a transfection control, pcDNA3-EGFP was an EGFP control, and HPRT was a negative control (26). Colony that posed superior amount of EGFP signal was chosen for next experiments with drug treatments. The transfections of pCBASceI, mCherry2-C1, and all other control plasmids were done. For the experiment with drug treatments, 300,000 HeLa cells per well were plated on six-well plates on day 1, followed by pCBASceI and control transfections on day 2 and drug treatment on day 3. Additionally, the repair assay was also analysed with live imaging with phase contrast and GFP channel (**Supplementary Tables S4, S6**).

### Apoptosis Assay

Total 100,000–200,000 cells were seeded per well in six-well plates, followed by drug treatment with vehicle, 5 µM IM, MX, and IMX. MX concentrations were 34 nM for HeLa, 103.5 nM for MDA-MB-231, and 9 nM for HCC-1937. Next, cells were incubated for 48 h. Cell pellets were collected and stained according to the kit protocol (**Supplementary Table S4**). Samples were then run with a flow cytometer. The data was analyzed with Flowing Software 2.5.1 (Mr Perttu Terho, Turku Bioscience Centre, Turku, Finland).

### Validation of Cytotoxicity of IMX *Versus* MX in Patient-Derived and Commercial HGSOC Cell Lines

Patient-derived cell lines including M022p, M022i, M048i, H002, and OC002 were plated with a seeding density of 5000–8000 per well in 96-wells plate. M022p, M022i, H002 and OC002 required the use of basement membrane matrix prior to plating (**Supplementary Table S18**). Dose preparation by serial dilution and IC_50_ measurement were done as mentioned before (see 2.4). Similar experimental design was applied to conventional cell lines including COV318, CaOV3, OVCAR3, OVCAR4, OVCAR5, COV362, Kuramochi, and OVCAR8.

### Statistical Analysis for Data Interpretation

Graph Pad Prism 6 was used in all experiments where P value of less than 0.05 was considered statistically significant. One-way ANOVA (Sidak’s multiple comparisons) was used to analyze more than two groups, and unpaired t-test was used to analyze only two groups in cell viability assay. Non-linear fit regression analysis (log inhibitor *vs*. response, extra sum-of-squares F-test) was performed to determine the IC_50_ value.

## Results

### Gene Set Enrichment Analysis (GSEA) Showed That IM Downregulates Pathways Related to DNA Repair in MX-Stressed Cells

We previously found that c-Abl inhibition after MX results in increased DNA cometing indicative of elevated DNA damage and massive cell death in cancer cells. Previously, we showed that inhibition of possible other IM substrates like c-kit and PDGF receptor kinase by AG1296 did not have any effect on cell viability. Moreover, c-Abl knockdown diminished the effect of IM in combination with MX in HeLa cells indicating its’ central role in IMX treatment (27). Thereby, we proposed the effect of IMX is due to inhibition of c-Abl in combination with topoisomerase II inhibition. Here, we show the mechanism in detail and therefore used HeLa cell line as model in RNA expression analyses. The primary objective of the microarray analyses was to determine the effect of c-Abl inhibition by IM in replicative stress conditions.

**Figure 1:**
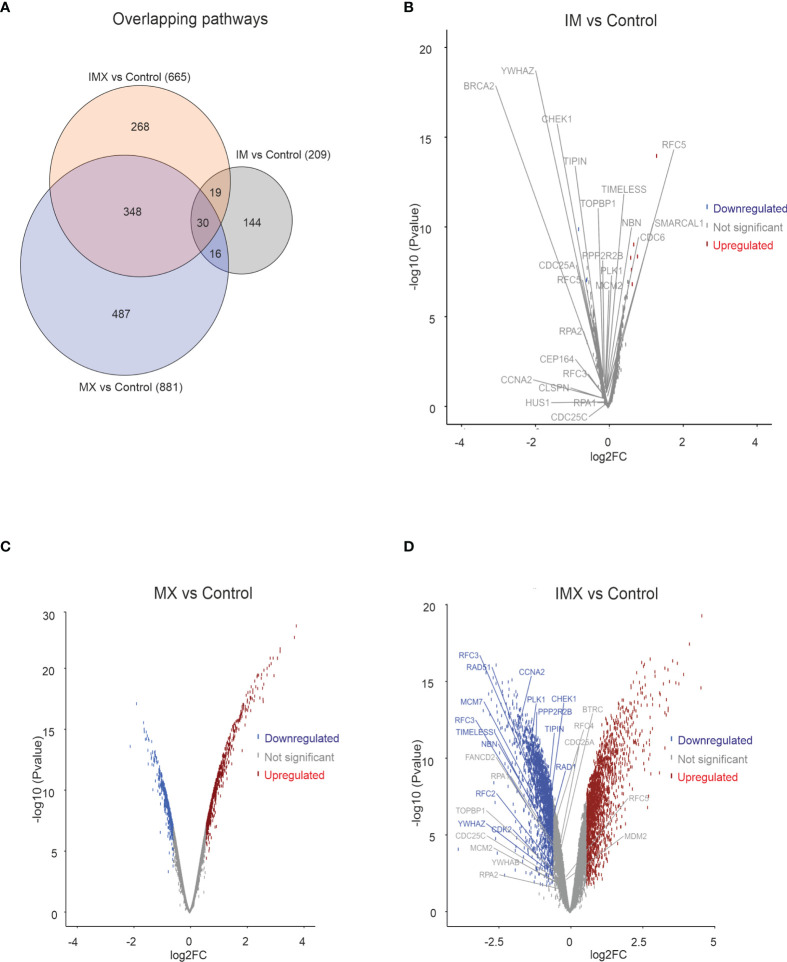
Transcriptome analysis in HeLa cell line. HeLa cells were treated with either imatinib (IM), mitoxantrone (MX), or IM + MX (IMX). The hybridization set consisting of 18196 differentially expressed genes was run against 18425 gene sets in a gene set enrichment analysis platform (GSEA). **(A)** Euler diagram showing the overlapping pathways. FDR q-value of 0.1 was used as a threshold. **(B–D)** Volcano plots of whole probe set with log2 fold change of 0.58 (FC = 1.5) of differentially expressed genes. Their significance values p = 0.01 were used as threshold. Red dots represent upregulated and blue dots downregulated genes. Dots below threshold are greyed. ATR pathway was downregulated in both IM *vs*. C and IMX *vs*. C, and the leading edge genes are separately depicted. None of the leading edge genes in IM-treated cells reached the threshold.

When IM was added to the vehicle, some gene pathways were enriched (**Figure 1A**) but the changes in individual gene levels were so subtle that only a few genes passed the threshold level of 1.5-fold increase and p < 0.05 (**Figure 1B**). The ATM-ATR pathway is central to the maintenance of genome integrity, and as an example, we depict the expression of leading edge genes of ATR pathway in both IM and IM+MX (IMX)-treated cells (**Figures 1B, D**). In the global expression analysis, only MX and IMX treatments resulted in significant up- or downregulation of genes, in contrast to IM (**Figures 1B–D**).

**Figure 2:**
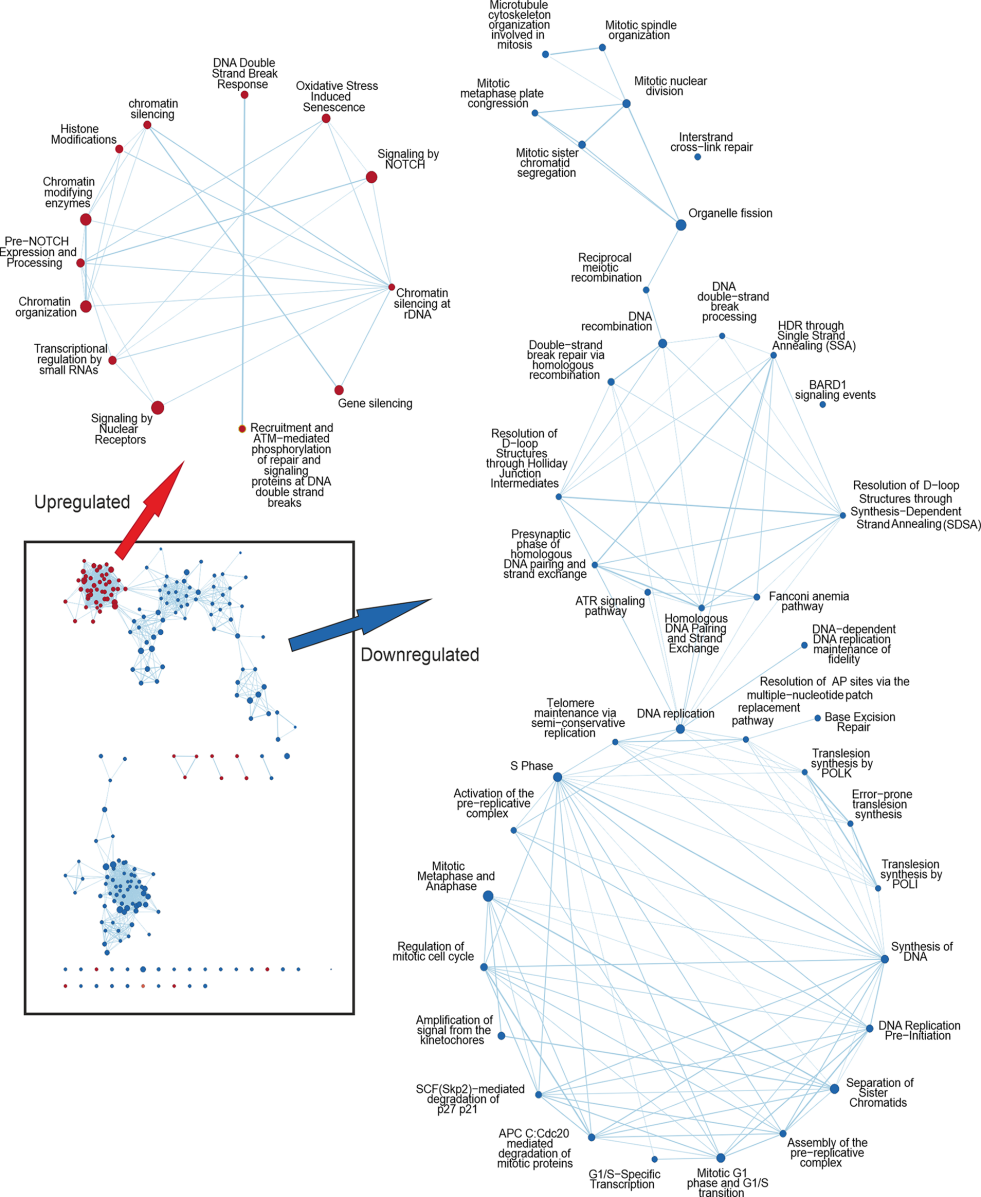
Cytoscape network analysis of the enriched pathways in IMX compared to MX indicating the effect of IM. Upregulated nodes in IMX are shown in red and downregulated nodes in blue. Lower left box represents the most significant nodes. Arrows depict the magnification of nodes filtered with diffusion algorithm. Pathways related to DNA damage response are upregulated, whereas pathways related to cell cycle progression and mitosis are downregulated. In the middle cluster of the downregulated nodes are pathways related to homologous recombination and ATR signalling.

GSEA analysis can detect subtle enrichment signals. The hundreds of pathways depicted in **Figure 1A** were clustered and visualized with Cytoscape (**Figure 2**). Compared to control, MX treatment yielded positive pathways which clustered in ‘metabolic pathways’, ‘stress-activated pathways’, and ‘mitochondrial pathway’ related to apoptosis, to mention the most significant.

Conversely, pathways regulating ‘chromatid separation and mRNA cytoplasmic translation’ were downregulated (data not shown). None of the stress-related pathways were detected in IM-treated samples. However, IM combined with MX (IMX) produced a new array of enriched pathways. The stress-signaling pathways, such as ‘interferon signaling, and p38 MAP-kinase stress cascade’ were also upregulated in IMX versus control as well as in IMX *vs*. MX. Similarly, ‘apoptosis and oxidative stress’ and ‘stress-induced senescence’ pathways were enriched in the upregulated phenotype. Several pathways were linked to ‘chromatin silencing and transcription repression’. Most notably, pathways related to HR and DNA repair were downregulated. Additionally, pathways involved in mitosis, like ‘microtubule organization’, ‘spindle formation’, ‘DNA replication maintenance’, ‘D-loop structure resolution’, ‘kinetochore amplification’, and ‘G1/S and mitotic metaphase/anaphase transitions’ were downregulated (**Figure 2** and **Supplementary Tables S7, S8**).

At the DNA damage sensor site, Reactome terms ‘recruitment and ATM-mediated phosphorylation of repair and signaling proteins at DNA double strand breaks’ and ‘DNA double-strand break response’ were upregulated in IMX *vs*. MX (**Figure 2**). ‘Non-homologous end joining’ was among the upregulated nodes in IMX *vs*. Control (FDR = 0.099), but did not reach the 0.1 FDR threshold in IMX *vs*. MX comparison. Pathways related to G1/S transition and especially G2/M were also downregulated.

Taken together, the transcriptome data suggest that addition of IM to non-stressed HeLa cells has very little, if any, effect on the pathway regulation, whereas the effect on MX-stressed cells is profound, especially for DNA repair. Defective genome maintenance can lead to many of the consequences seen in the pathway enrichment analysis and necessitates further, direct functional scrutiny.

### IM Reduces IC_50_ of MX in Cancer Cells

**Figure 3:**
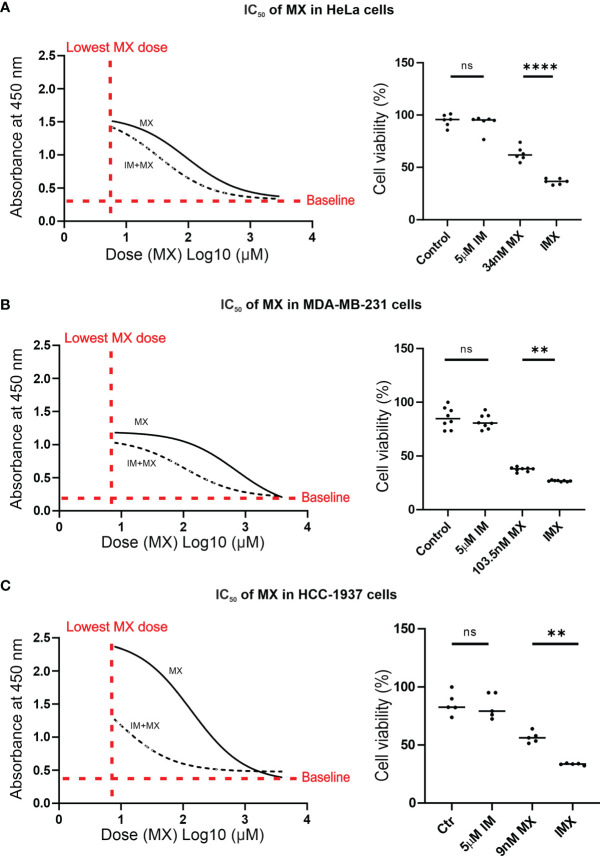
IM reduces IC50 of MX in cancer cells. In A–C, the non-linear plots show dose *vs*. response curves where X-axis presents dose (MX) Log10 (µM) and Y-axis represents absorbance at 450 nm, which is proportional to the amount of viable cells (p < 0.05). The column scatter plots represent median cell viability (%), where the effect of MX combined with 5 µM IM (IMX) is compared to 0.1% DMSO (control), 5 µM IM, and MX (ordinary one-way ANOVA: p < 0.05). **(A)** In HeLa cells, IC_50_ of MX was 92.5 nM (95% confidence interval (CI): 63–136.5), and IC_50_ of MX combined with 5 µM IM (IMX) was 34 nM (95% CI: 25–46). **(B)** In MDA-MB-231 cells, IC_50_ of MX was 707 nM (95% CI: 542.5–921) and IC_50_ of MX with 5 µM IM (IMX) was 103.5 nM (95% CI: 86–124). **(C)** In HCC-1937 cells, IC_50_ of MX was 136 nM (95% CI: 104.5–176) and IC_50 of_ MX with 5 µM IM (IMX) was 9 nM (95% CI: 5–16). **p<0.005, ****p<0.0001; ns, non-significant.

In the microarray experiments, we used 0.8 µM MX, 5 µM IM and the combinations as initial drug concentrations for HeLa cells. For all subsequent functional analyses, MX IC_50_ values were determined for the cell lines used (HeLa, MDA-MB-231, and HCC-1937) (**Figure 3**). We also used FUCCI-HeLa for which HeLa IC_50_ was utilized. HeLa, FUCCI-HeLa, and MDA-MB-231 cells were validated by panel sequencing of HR-related genes to exclude possible alterations in other DNA repair pathways at the DNA level (data not shown). The HCC-1937 cell line is BRCA1-mutated (28, 29). In line with our previous studies with cervical and vulvar cancer cell lines (27), IM treatment alone did not reduce cell viability. We found that 5 µM IM significantly reduced viability of MX-treated HeLa, MDA-MB-231, and HCC-1937 cells regardless of their HR status (**Figure 3** and **Supplementary Figure S9**). IMX (5 µM IM + 34 nM MX) showed similar effect as 92.5 nM MX in HeLa cells, 5 µM IM + 103.5 nM MX showed similar effect as 707 nM MX in MDA-MB-231 cells, and 5 µM IM + 9 nM MX showed similar effect as 136 nM MX in HCC-1937 cells.

### Imatinib Increases DNA Damage But Reduces Nuclear RAD51 Levels

**Figure 4:**
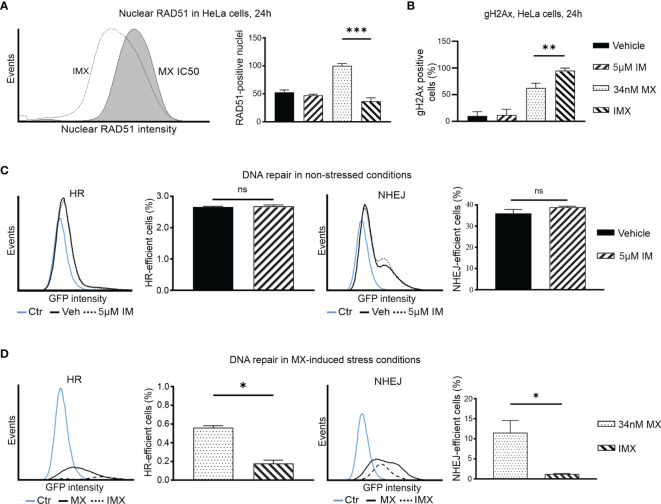
IM decreases nuclear RAD51 level after DNA-damage and blocks DNA repair in cancer cells. **(A)** Histogram shows nuclear RAD51 intensity under 34 nM MX and 5 µM IM + 34 nM MX (IMX) treatment. The bar chart shows percent of nuclear RAD51 population after different treatments (95% CI of difference for MX *vs*. IMX: 47.49–82.51 where p = 0.0004, ordinary one-way ANOVA). **(B)** Bar chart shows percentage of γH2Ax-positive cells (95% CI of difference for MX *vs*. IMX: -54.26 to -10.56 where p = 0.0063, Ordinary one-way ANOVA). **(C)** The histograms show HR and NHEJ repair signal intensity, and bar chart shows percent of HR and NHEJ-efficient cells (corrected against transfection efficiency) after IM treatment in non-stressed conditions (95% CI of difference for HR: 5 µM IM *vs*. vehicle: -0.1189–0.1662 where p = 0.5499, NHEJ: 5 µM IM *vs*. vehicle: -2.764–8.630 where p = 0.1571, two-tailed, unpaired t-test). **(D)** The histograms show HR and NHEJ repair signal intensity, and bar chart shows percent of HR and NHEJ-efficient cells (corrected against transfection efficiency) after IM treatment in MX-induced stress conditions (95% CI of difference for HR: 5 µM IM *vs*. vehicle: -0.5037 to -0.2601 where p = 0.0054; NHEJ: MX *vs*. IMX: -19.49 to -1.139 where p = 0.0402, two-tailed, unpaired t-test). *p<0.05, **p<0.005, ***p=0.0001; ns, non-significant.

Pathway analysis (**Figure 2**) revealed that IMX treatment resulted in upregulation of the DSB response but downregulation of homology-directed recombination repair pathways (**Figure 2**). We tested whether these putative changes were directly detectable in drug-treated cells. To this end, we used flow cytometry (**Figure 4A**) and immunocytochemistry (**Supplementary Figures S10–S12**). The expression of γH2AX, a marker for DNA damage, increased after MX treatment, and IMX caused a more pronounced increase of DNA damage. IM alone did not show significant effect compared to vehicle (**Figure 4B** and **Supplementary Figures S10–S13**).

We further studied RAD51 expression as a proxy for HR-mediated DNA repair. Isolated HeLa cell nuclei were analyzed using flow cytometry after 24 h of drug treatment (30). MX-induced stress resulted in increase in RAD51 levels, indicative of augmented HR. However, further increase in DSBs after IMX treatment did not result in a corresponding accumulation of nuclear RAD51 levels, but instead a clear attenuation of the signal at 24 h after treatment (**Figure 4A**). This suggests that IMX prevented nuclear localization of RAD51.

Similarly, using indirect immunofluorescence, we observed a marked increase in RAD51 signal after MX treatment, but diminished amount of RAD51 foci in IMX-treated MDA-MB-231 cells (**Supplementary Figure S11**). When BRCA1 was silenced with siRNA, IMX increased γH2AX levels and reduced RAD51 levels (**Supplementary Figure S12**). This suggests that c-Abl may directly facilitate RAD51 activity independently of BRCA1 after DNA damage, in line with previous studies.

### HR and NHEJ Are Suppressed by Imatinib After DNA Damage

To study DNA repair, we used two HeLa cell lines that contain HR or NHEJ reporter cassettes expressing GFP when DNA repair occurs (**Figure S14**). The baseline HR efficiency of HeLa cells was 0.03 and the baseline NHEJ efficiency of HeLa cells was 0.12, four times higher than HR (**Supplementary Figure S15**). Treatment with IM alone did not alter DNA repair activity (**Figure 4C**). In **Figure 4C**, vehicle and 5 µM IM treatment lines overlap suggesting no difference in HR or NHEJ capacity of the cells in the absence of stress. In contrast, GFP signal was lower and fewer cells were positive with IMX compared to MX (**Figures 4C, D**). Thus, IM significantly suppressed both HR and NHEJ in MX-damaged cells. The findings in the DNA repair reporter assays are consistent with the GSEA data and with the observed RAD51 data, as RAD51 is responsible for the actual recombination step in HR (**Figure 4A**). Interestingly, in line with this, our microarray data suggested that expression of the PRKDC gene, encoding a critical NHEJ component DNA-PK, was 2.3-fold downregulated in IMX-treated cells compared to vehicle (p = 2.7 x 10^-6^, FDR adj. p = 1.8x10^-5^).

**Figure 5:**
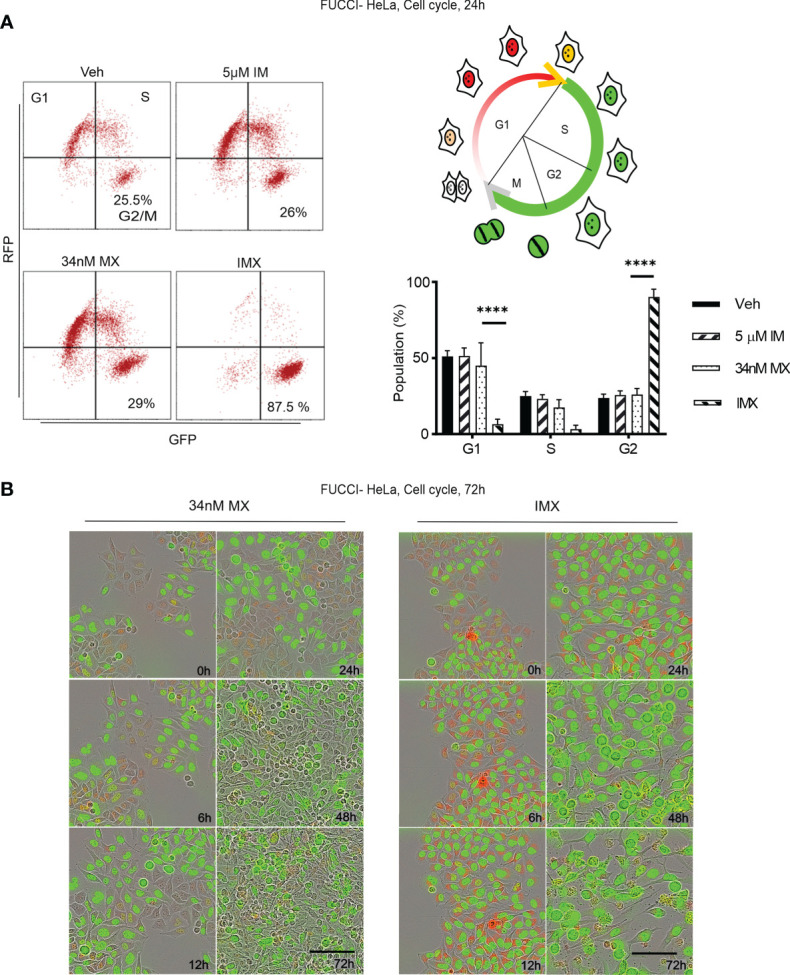
c-Abl inhibition in combination with MX causes complete G2 arrest in FUCCI-HeLa cells. **(A)** Flow cytometry data on FUCCI-HeLa cells in different cell cycle phases. The representative dot plots show cell cycle after different treatments where lower right quadrant shows G2/M population. The picture demonstrates cell cycle in terms of FUCCI system where newly divided daughter cells have no color, G1 cells show red fluorescence, G1-S transition shows yellow fluorescence, and S/G2/M shows green fluorescence. G2/M population was separated from S in FACS analysis. Bar chart shows percent of population in each phase (95% CI of difference for G0/G1 phase: MX *vs*. IMX is 21.88–55.45 where p < 0.0001; G2/M phase: MX *vs*. IMX is -82.89 to -45.35 where p < 0.0001, 2 way ANOVA). **(B)** Representative images of FUCCI-HeLa cells in 5 time points after 34 nM MX or IMX treatments. Scale bar = 50 µm. ****p<0.0001.

We further followed the temporal and functional course of DNA repair in MX-treated HeLa cells by time-lapse microscopy for 7 days. GFP-expressing cells with a HR reporter cassette underwent normal bipolar cell division in the presence of MX (**Supplementary Video S17**). After cell division, these cells entered G1 normally, which is consistent with flow cytometry analysis, where a distinct G1 population was present after MX treatment (**Figure 5**). For the GFP-expressing cells with a NHEJ reporter cassette, normal metaphase was observed after MX treatment. There, cells were able to employ NHEJ and normal cell division ensued (**Supplementary Video S17**).

Collectively, this suggests that IM impairs both HR and NHEJ-mediated repair of DNA damage in cells with topoisomerase II inhibition-induced replication stress.

### Imatinib Induces G2/M Arrest and Apoptosis in MX-Treated Cancer Cells

According to pathway analysis, IMX treatment downregulates pathways such as ‘S-phase’, ‘synthesis of DNA’, ‘G2/M transition’, ‘G1/S transition’, and ‘mitotic cell cycle’. For functional validation, we studied cell cycle using FUCCI-HeLa cells. The fusion protein mKO-Ctd1, expressed in G1, confers cells in this phase red fluorescence, while mAG-Geminin, expressed throughout S-M phase, produces green fluorescence. Cells transitioning from G1 to S show yellow fluorescence, while right after cell division no fluorescence can be detected (31). After MX treatment, these cells were able to enter mitosis after prolonged G2 (**Supplementary Video S18**). Flow cytometry data showed no G2/M arrest after MX treatment (**Figure 5A**). In sharp contrast, with IMX there was a delay in G1/S progression and a population of FUCCI-HeLa cells with red fluorescence accumulated in G1 for 6–8 h (**Figure 5B**). Eventually, cells exited from G1, entered S phase, and finally the entire cell population accumulated in G2/M phase with green fluorescence and larger nuclear size (data not shown). Moreover, cells which accumulated in G2/M phase did not enter mitosis, and eventually died over the 7-day observation period. The complete G2/M block was verified by flow cytometry (**Figure 5A**).

**Figure 6:**
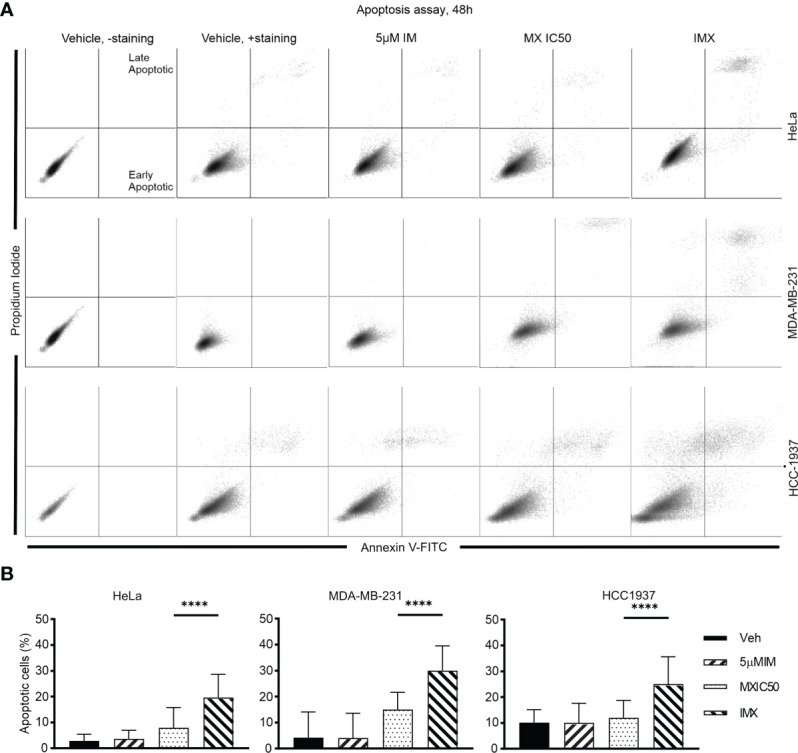
IM induced apoptosis in HeLa, MDA-MB-231, and HCC-1937 cells after DNA damage. **(A)** The dot plots show distribution of early and late apoptotic populations in different cell lines. Annexin V-positive cells in the lower right quadrant are early apoptotic. Double-positive population in the upper right quadrant is late apoptotic/necrotic. The percentages of both early and late apoptotic populations are calculated as total percent of apoptotic population. **(B)** The bar chart shows the percent of apoptotic cells in total population under different treatments in different cell lines. ****p<0.0001.

In addition, G2/M arrest was also seen in MDA-MB-231 and HCC-1937 cells after IMX treatment (**Supplementary Figure S16**). According to time-lapse microscopy, apoptosis followed by G2/M arrest was the hallmark of IMX effect, which implies that IMX-treated cells were unable to repair DNA damage (**Supplementary Video S18**). There was also a statistically significant increase in the apoptotic cell population in HeLa, MDA-MB-231, and HCC-1937 cell lines after IMX treatment (**Figure 6**). Although there was upregulation of ‘oxidative stress-induced senescence’ after IMX treatment in micro-array analysis (**Figure 2**), we observed that cancer cells die predominantly by apoptosis after G2/M arrest.

### Imatinib Significantly Reduces Viability of Patient-Derived and Commercial HGSOC Cells After MX Irrespective of Their HR Status

HR proficiency is a clinical problem that is centrally linked to development of resistance to treatments. Therefore, we wanted to test if the effect on cytotoxicity in different adenocarcinomas and squamous cell cancers is also applicable to HGSOC.

We validated the effect of c-Abl inhibition on MX-induced lethality in five patient-derived and eight commercial HGSOC cell lines. **Supplementary Tables S19, S20** show that all the HGSOC cell lines have known HR-status. Cell viability assay was conducted to determine the IC_50_ of MX ( ± IM). These cell lines were not sensitive to IM. Therefore, there is no IC_50_ for IM.

**Figure 7:**
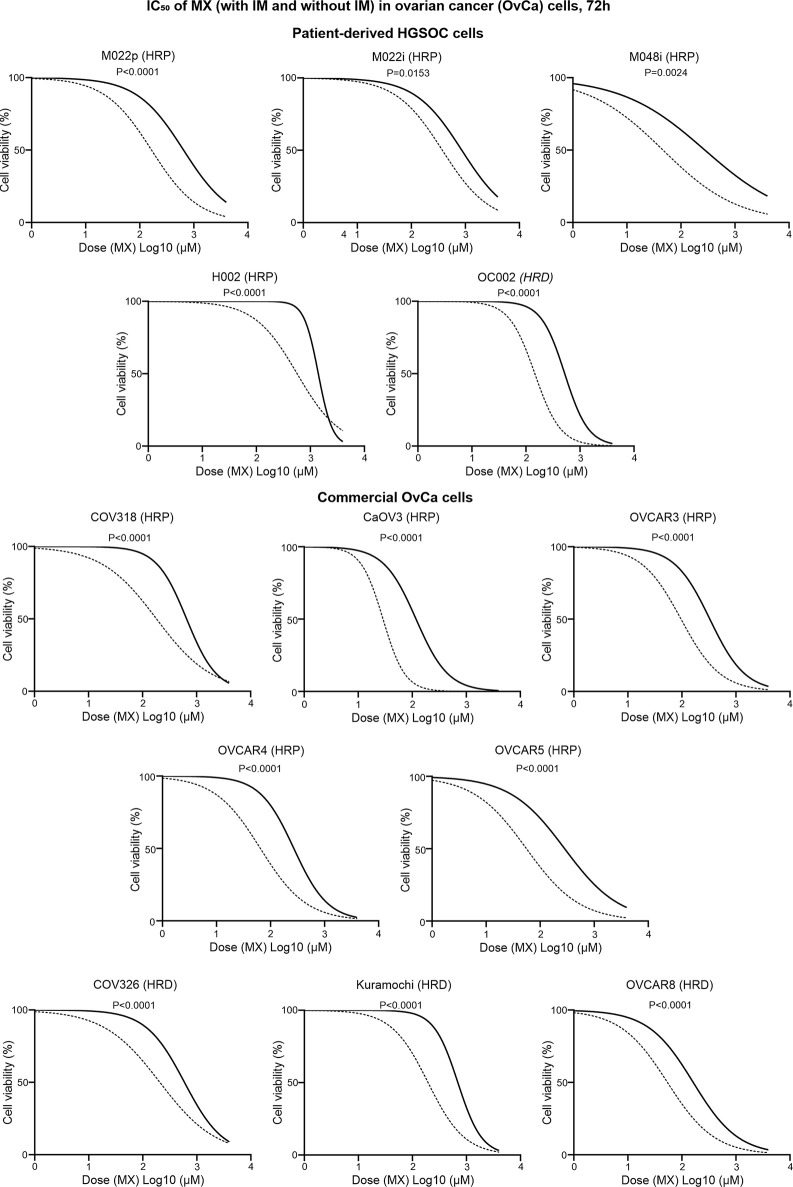
IC_50_ values of MX and IMX in ovarian cancer cell lines after 72 h of treatment. The cell lines include five patient-derived HGSOC cell lines (M022p, M022i, M048i, H002, and OC002) and eight commercial HGSOC cell lines (COV318, CaOV3, OVCAR3, OVCAR4, OVCAR5, COV326, Kuramochi, and OVCAR8) with known HR status (either HRP or HRD). The non-linear regression plots show log (inhibitor) *vs*. normalized response-variable slope where black solid line is MX-only (without IM) and dotted line is MX with 5 µm IM.

M022p, M022i, M048i, and H002 are patient-derived HRP HGSOC cells. M022p is obtained from laparoscopy at diagnosis. M022i, M048i, and H002 are obtained from patients during interval surgery after three courses of neoadjuvant chemotherapy treatment. OC002 is HRD cell line obtained from patient with primary laparoscopy at diagnosis (**Supplementary Table S19**). IC_50_ data shows that IM decreased IC_50_ MX-dose by 74.5%, 56%, 83%, and 59.5% in M022p, M022i, M048i, and H002 cell lines, respectively. In OC002 cells, MX-dose decreased by 72% after IM combination (**Supplementary Table S19**). We also tested HRP HGSOC commercial cell lines COV318, CaOV3, OVCAR3, OVCAR4, and OVCAR5. CaOV3, which has known platinum resistance, was unexpectedly the most sensitive to MX (**Supplementary Table S20**). In contrast, COV318 with known platinum resistance, was the least sensitive to MX among all HRP cell lines (**Figure 7** and **Supplementary Table S20**). Additionally, we studied COV362, Kuramochi and OVCAR8 HRD cell lines. Kuramochi was the least sensitive to MX though it has been reported to be platinum sensitive (**Supplementary Table S20**). In all these cell lines regardless of HR-status, neoadjuvant chemotherapy, or platinum-sensitivity, IM significantly reduced the IC_50_ dose of MX. The results also show that the previously reported platinum sensitivities do not directly correlate with mitoxantrone toxicities.

## Discussion

During carcinogenesis, neoplastic cells often lose a critical DDR pathway. This is an Achilles heel of cancers with high replication stress, which has been exploited with radiation therapy and DNA-targeting chemotherapy (32). Examples of the latter include topoisomerase I and II inhibitors that prevent decatenation and resealing of chromosomal breaks inducing unresolved DSBs during replication (33, 34). DSBs are detrimental to the cell due to the need of accurate chromosome segregation during mitosis, but importantly also due to persistent DDR signalling. DSBs activate DDR *via* ATM-dependent manner to delay cell cycle progression to facilitate repair. Successful high-fidelity DSB repair involves HR. HR defects, e.g., due to inactivating mutations in *BRCA1* and *BRCA2* genes, are exploited in ovarian and breast cancer treatment, where single strand repair inhibitors (PARP inhibitors) as post-chemotherapy maintenance treatment show high efficacy in the HR-deficient subpopulation of patients. In contrast, HR-proficient tumours are usually resistant to PARP inhibitors and to chemotherapy in general (35, 36). Here, we show that HR can be blocked with simultaneous treatment of mitoxantrone and imatinib.

In this study, we used γH2AX as a marker for DNA damage and found increased nuclear signal after MX treatment, which was further augmented with IM in all tested cell lines, regardless of their BRCA1 status. We studied the pathway-level effects of DSB accumulation in HeLa cells at the global transcriptomic level with useful hints for further mechanistic analyses which is consistent with Peng and co-workers who showed that HR deficiency and protein expression levels closely correlated with changes at the transcriptional level (37). We found that c-Abl inhibition after MX treatment downregulated several DNA repair pathways, most notably those related to HR. In the network analysis, these nodes were connected to downregulation of cell cycle transition in G2/M and mitosis. Limited availability of dNTPs causes replication fork stalling (38). We found deoxyribonucleotide triphosphate (dNTP) synthesis among the downregulated pathways, providing a potential explanation for replication stress. Collectively, gene expression analysis suggested broad impairment of DNA repair in response to IMX, which we studied in more detail.

There is a constitutive interaction between the PXXP motif in the C-terminus of BRCA1 and the SH3 domain of c-Abl (16). This type of interaction is disrupted in an ATM-dependent manner after irradiation, followed by increased c-Abl kinase activity. In addition, c-Abl facilitates the progression of HR by activating RAD51, and allows its translocation into the nucleus after DNA damage (13, 39, 40). In the present study, we found that MX increased nuclear RAD51 levels in BRCA1-wt HeLa and MDA-MB-231 cells. However, RAD51 levels were significantly higher under MX treatment compared to vehicle even after BRCA1 silencing. Adding IM to MX significantly reduced nuclear RAD51 expression in Hela, BRCA1-wt MDA-MB-231, and BRCA1-silenced MDA-MB-231 cells. These findings suggest that the effects of concurrent inhibition of c-Abl and topoisomerase II are independent of BRCA1 status and depict a major role for c-Abl in DNA repair in these cancer cells. Moreover, BRCA1 is considered as negative regulator of c-Abl. BRCA1-mutant cancer cell line HCC-1937 has a constitutively high c-Abl kinase activity (16). This suggests a dependency on c-Abl, which is supported by the finding that HCC-1937 was the cell line most sensitive to IMX, with a 15-fold decrease in IC_50_ compared to MX.

The aforementioned findings are supported by gene expression data showing downregulated HR in response to IMX. Correspondingly, in a direct HR reporter assay, significantly reduced HR was observed in IMX-treated HeLa cells. The other major DSB repair pathway, NHEJ, does not rely on the presence of the replicated sister chromatid (41). We therefore asked whether loss of HR could be compensated by NHEJ. The fusion form of c-Abl (BCR-Abl) upregulated NHEJ in chronic myeloid leukaemia after irradiation suggesting that IM may reduce NHEJ (17). The regulation of NHEJ by c-Abl in solid cancers remains poorly understood. In this study, we found that IM reduces NHEJ capacity in HeLa cells.

Of note, HeLa cells used in the experiments express HPV18 E6 which binds and degrades tumor suppressor p53, and still show dramatic stabilization of p53 with a 4-fold reporter activation in IMX (27). Our previous study also showed that p53 reactivation is possible after treatment despite destabilization by E6 (42, 43). By transcriptome profiling in this study, we also detected a 4.5-fold increase in *CDKN1A* (p21) which is transcriptionally regulated by p53. The ATM response pathway which directly phosphorylates p53 was upregulated in GSEA after IMX treatment. In addition, ATM kinase is a major physiological mediator of H2AX phosphorylation in response to DSBs, producing γH2AX, and this was highly elevated in IMX-treated cell lines. Taken together, our findings indicate that the DNA damage sensor site is working in these cells, but the effector part of the downstream kinase cascade is tilted towards DDR inhibition, persistent cell cycle arrest, and eventual death. The previous study showed that loss of c-Abl may cause senescence in mouse embryonic fibroblasts (44). We also observed upregulation of ‘oxidative stress-induced senescence’ in microarray data but the apoptosis assay and time-lapse microscopy suggests predominant death of cancer cells *via* apoptosis after G2/M arrest.

Inactivating *BRCA1* and *BRCA2* mutations result in HR dysfunction that occurs in 50% of HGSOC (35). Though BRCA1/2*-*deficient cancers are sensitive to platinum-based therapy, secondary *BRCA1/2* mutations are also responsible for acquired resistance to cisplatin in ovarian carcinomas (45, 46). In BRCA-mutated tumors, synthetic lethality can be induced by PARP inhibitors (47). PARP inhibitor efficacy relies heavily on tumor HR deficiency, and PARP inhibitors have limited or no activity in tumors with functional HR (36). So ideally, inducing HR deficiency in resistant tumors would make them sensitive to DNA-damaging chemotherapy and to PARP inhibitors. This was achieved in HRP epithelial ovarian cancer, which is sensitive to PARP inhibitors when combined with PI3K inhibitor alpelisib that inhibits HR (48). Interestingly, PI3K is downstream to Abl in the HR pathway (49, 50). In the present study, we used MDA-MB-231 cell line with different BRCA1 statuses to assess whether the IMX treatment outcome is dependent on the function of this protein which is essential for HR. Neither cell viability, cell cycle distribution, γH2Ax expression, nor nuclear RAD51 levels were different in parental MDA-MB-231 cells with intact BRCA1, compared to BRCA1-silenced cells after IMX treatment. We also observed that IMX has similar cytotoxic effect on cell viability of HeLa, MDA-MB-231, HCC-1937, and 13 HGSOC cell lines, which are squamous cell and adenocarcinoma. Among these cell lines, in addition to HeLa and MDA-MB-231, 11 cancer cell lines have functional BRCA1 and HR. We used three commercial BRCA1-mutant HRD HGSOC cell lines, which may have high c-Abl activity similar to HCC-1937 (16). HeLa was used as a cancer cell model for DNA repair assays. Other *in vitro* assays were performed in MDA-MB-231 and HeLa, where IMX caused reduction of nuclear RAD51 and HR-proficiency. It is likely that same mechanism is responsible for the lethality of IMX on HRP HGSOC cell lines where similar cytotoxicity was achieved. In HeLa cells we showed that IMX-treated cells have reduced NHEJ-capacity. As it is known that HRD cancer cells mostly rely on NHEJ, IMX may also work *via* this mechanism in HRD HGSOC cells. Moreover, our data suggested that IMX is lethal to HGSOC cell lines, both patient-derived and commercial, regardless of their HR-status and platinum-sensitivity.

Despite the therapeutic success of PARP inhibitors in HRD cancers, several other DDR targets have gained interest. For example, inhibitors of ATR, ATM, DNA-PK, CHK1 and 2, and WEE1 are in clinical trials. Efficacy of single-agent use is dependent on and often hampered by the tumors’ proficiency to deal with different forms of DNA damage. Adding chemotherapy to these compounds, or combining them with different DDR inhibitors may increase toxicity substantially (51). Although we do not know the potential clinical toxicity of the present IMX combination, it is noteworthy that the cell lines here or in our previous report were rather inert to IM in non-stressed condition. The finding that IM increases the therapeutic index of MX 3- to 15-fold suggests that the concentration of this compound can be reduced several fold, resulting potentially to fewer side effects of which cardiomyopathy can lead to severe consequences in long term use (52). In this respect, it is reassuring that even a long-term administration of IM to chronic myeloid leukemia patients does not cause unacceptable cumulative or late toxic effects (53). We also have previously tested IMX in normal low-passage fibroblasts and found that IM did not alter the proliferation of these cells when combined with MX, suggesting that malignant cells are more vulnerable to IMX than normal cells (27).

Overall, our results demonstrate that c-Abl inhibition increases the effect of topoisomerase II inhibitor MX. Moreover, the data indicate that both HR and NHEJ are suppressed by this dual inhibition, potentially opening up a possibility for better efficacy even in hard-to-treat HR-proficient cancers.

## Conclusions

This study provides evidence that concomitant c-Abl and topoisomerase II inhibition suppresses HR-mediated DSB repair. Additionally, the combination treatment may also suppress NHEJ-mediated DSB repair, leading to G2 arrest, and eventually apoptosis of cancer cells. We also demonstrate that addition of IM to MX significantly increases the therapeutic index of MX and shows strong synergy in killing of patient-derived HGSOC cell lines, independent of BRCA1 status.

## Data Availability Statement

The datasets presented in this study can be found in online repositories. The names of the repository/repositories and accession number(s) can be found below: https://www.ebi.ac.uk/arrayexpress/, E-MTAB-9475.

## Author Contributions

AS and SH conceived the project and designed experiments. AS carried out experiments, analysis, and interpretation of data and prepared original draft. MT conducted immunofluorescence experiment. AJ conducted FUCCI-HeLa cell cycle assay with FACS. JS and SA visualized data and provided technical guidance to AS. RV performed DNA sequencing. KK and PR developed, maintained, and provided patient-derived HGSOC cell lines for conducting assays. SH, LK, MT, SA, and KH revised the manuscript. All authors reviewed the article. SH, AS, and JS finalized the manuscript. All authors contributed to the article and approved the submitted version.

## Funding

This work was funded by the Finnish Cancer Foundation and the Sigrid Jusélius Foundation (grants to LK) and the Academy of Finland (grant number 314338 to SH, 308375 to JT, and 314394 to LK).

## Conflict of Interest

The authors declare that the research was conducted in the absence of any commercial or financial relationships that could be construed as a potential conflict of interest.

## Publisher’s Note

All claims expressed in this article are solely those of the authors and do not necessarily represent those of their affiliated organizations, or those of the publisher, the editors and the reviewers. Any product that may be evaluated in this article, or claim that may be made by its manufacturer, is not guaranteed or endorsed by the publisher.
