# Supplementary material for: Targeting DNA Homologous Repair Proficiency With Concomitant Topoisomerase II and c-Abl Inhibition
Source: Front Oncol. 2021 Sep 20;11:733700. doi: 10.3389/fonc.2021.733700 (PMC8488401; doi:10.3389/fonc.2021.733700)
Supplement: Supplementary file 1 [file DataSheet_1.pdf]

Supplementary Table S1: List of commercial cell lines with sources and research resource identifiers (RRIDs)

<table>
<tr><th>Cell lines</th><th>Cancer type</th><th>Source</th><th>Details</th></tr>
<tr><td>HeLa</td><td>Cervical cancer</td><td>ATCC</td><td rowspan="3">Mutation analysis to confirm BRCA1-status of cell lines was performed using an in-house Ampliseq panel (Huslab laboratory, Helsinki, Finland) and the Ion Torrent sequencing platform. The panel includes following genes: BARD1, BLM, BRCA1, BRCA2, BRIP1, CDH1, CHEK2, DICER1, FANCM, MCPH1, MLH1, MSH2, MHS6, PALB2, PMS2, PTEN, RAD51C, RAD51D, STK11, TP53, and four known Finnish germline mutations of the ATM-gene.

HeLa (RRID:CVCL_0030)
HeLa/Fucci (RRID:CVCL_E070)
MDA-MB-231 (RRID:CVCL_0062)</td></tr>
<tr><td>FUCCI-HeLa</td><td>Cervical cancer</td><td>RIKEN BioResource Research Center Cell Bank, Ibaraki, Japan</td></tr>
<tr><td>MDA-MB-231</td><td>Triple-negative breast cancer (TNBC)</td><td>ATCC</td></tr>
<tr><td>HCC1937</td><td>Triple-negative breast cancer (TNBC)</td><td>ATCC</td><td>HCC1937 (RRID:CVCL_0290)</td></tr>
<tr><td>COV318</td><td>HGSOC</td><td>ATCC</td><td>COV318 (RRID:CVCL_2419)</td></tr>
<tr><td>CaOV3</td><td>HGSOC</td><td>ATCC</td><td>Caov-3 (RRID:CVCL_0201)</td></tr>
<tr><td>OVCAR3</td><td>HGSOC</td><td>ATCC</td><td>OVCAR-3 (RRID:CVCL_0465)</td></tr>
<tr><td>OVCAR4</td><td>HGSOC</td><td>ATCC</td><td>OVCAR-4 (RRID:CVCL_1627)</td></tr>
<tr><td>OVCAR5</td><td>HGSOC</td><td>ATCC</td><td>OVCAR-5 (RRID:CVCL_1628)</td></tr>
<tr><td>COV362</td><td>HGSOC</td><td>ATCC</td><td>COV362 (RRID:CVCL_2420)</td></tr>
<tr><td>Kuramochi</td><td>HGSOC</td><td>ATCC</td><td>Kuramochi (RRID:CVCL_1345)</td></tr>
<tr><td>OVCAR8</td><td>HGSOC</td><td>ATCC</td><td>OVCAR-8 (RRID:CVCL_1629)</td></tr>
</table>

Supplementary Table S2: List of patient-derived cell lines with source

Cell lines	Source	Details
M022p	TUH	The patient specific p53 mutations from the established cell lines were confirmed by sequencing. Mutational profile including SBS3 signature was obtained. Functional HR-score was also calculated by quantifying the percent of HR-proficient cells among epithelial cells in G2. The same was done for commercial cell lines including COV318, CaOV3, OVCAR3, OVCAR4, OVCAR5, COV362, Kuramochi and OVCAR8.
M022i	TUH	
M048i	TUH	
H002	TUH	
OC002	TUH	
TUH= Turku University Hospital		

Supplementary Table S3: Details of culturing commercial and patient-derived cell lines.

Cell culture media and growth condition	
Commercial cell lines	Patient-derived cell lines
All commercial cell lines were cultured in DMEM (Sigma-Aldrich, Missouri, USA) or RPMI-1640 (Sigma-Aldrich, Missouri, USA) supplemented with 10% heat-inactivated fetal bovine serum (FBS), 1% non-essential amino acids, 1% L-glutamine (Gibco®, Life technologies, UK) and 1% penicillin-streptomycin (Sigma-Aldrich, Germany). OVCAR3 and OVCAR8 cell lines required 10µg/ml insulin.	All patient-derived adherent cell lines were cultured in RPMI-1640 (Sigma-Aldrich, Missouri, USA) supplemented with 10-20% FBS, 100µg/ml penicillin-streptomycin and 2mM ultraglutamine (Lonza). Spheroidal cell culture medium DMEM-F12 (Lonza) was supplemented with 20ng/ml EGF (Gibco life technologies), 10ng/ml bFGF (Gibco life technologies) and 1x B27 (Gibco life technologies).

Cell lines were grown at 37°C in a humidified atmosphere with 5% CO_2, and regularly tested for mycoplasma.

Supplementary Table S4: List of reagents and consumables in laboratory experiments.

Materials and methods sections	Reagents and consumables	Catalog & source
4.1	Greiner CELLSTAR® 6 well plates	657160, Germany
	Histopaque-1077	10771-100ML, Sigma-Aldrich, Germany
	DMSO	SC-358801, ChemCruz, SantaCruz Biotechnology, USA
	Mitoxantrone	M6545-10MG, Sigma-Aldrich, Germany
	Imatinib mesylate	SML1027-10MG, Sigma-Aldrich, Germany
4.2	Trizol RNA isolation reagents	15596026, ThermoFisher scientific, USA
	RNeasy Mini kit	74104, Qiagen, Hilden, Germany
4.4	Greiner CELLSTAR® 96 well plates	M0812-100EA, Germany
	CCK-8 kit	CK04, Dojindo Molecular, USA
4.7	NE-PER™ Nuclear and cytoplasmic extraction reagents	78833, ThermoFisher scientific, USA
4.8	Click-iT™ EdU Alexa Fluor™ 488 Flow Cytometry Assay Kit	C10425, ThermoFisher scientific, USA
	Fixation Buffer BD CytoFix ®	BD 554655, Fisherscientific, USA
4.9	FlexiTube siRNA BRCA1	siRNA Name: Hs_BRCA1_13, cat. no.: SI02654575 and Hs_BRCA1_14, cat. no.: SI02664361, QIAGEN, USA
	Lipofectamine® RNAiMAX	(#13778030, Life Technologies Europe
	Pierce™ Protease Inhibitor Mini Tablets, EDTA-free	A32955, Thermo Scientific, USA
	Pierce™ BCA Protein Assay Kit	23225, Thermo Scientific, USA
4.10	Hoechst 33342	H3570, Thermo Scientific, USA
	ProlongGold anti-fade mounting media	P36930, Thermo Scientific, USA

4.11	Plasmid linearization reagent XhoI (10 U/µL)	ER0691, ThermoFisher Scientific, USA
	Plasmid transfection reagent Lipofectamine 3000	L3000001, ThermoFisher Scientific, USA
4.12	Annexin V-FITC Apoptosis Staining / Detection Kit	ab14085, Abcam
4.13	Basement membrane matrixGeltrex™, Gibco™	12063569, ThermoFisher Scientific, USA

Supplementary Table S5: List of all primary and secondary antibodies.

Primary and secondary Antibodies	Remarks	
Rabbit Anti-Rad51 antibody [EPR4030(3)]	Dilution 1:50, ab133534, Abcam	
Mouse Anti-BRCA1 mAB	Dilution 1: 50, OP92, Calbiochem®, Germany	
Mouse α-γH2Ax	Dilution 1:1000, ab22551, Abcam, UK	
Rabbit α-RAD51	Dilution 1:500, sc-8349, Santa Cruz Biotechnology, Texas, USA 1:500	
Rabbit α-cleaved Caspase-3	Dilution 1:300, 9664 Cell Signaling Technology, Massachusetts, USA	
Goat actin	Dilution 1:100, sc-1616, Santa Cruz Biotechnology, Texas, USA, UK	
Donkey anti-mouse IgG-Alexa Fluor 488	A21202	Invitrogen, California, USA; all diluted 1:1000
Donkey anti-goat IgG-Alexa Fluor 488	A32814	
Donkey anti-mouse IgG-Alexa Fluor 568	A31571	
Donkey anti-rabbit IgG-Alexa Fluor 647	A11057	
Donkey anti-rabbit IgG-AlexaFluor 488	A21206	

Supplementary Table S6: List of all plasmids with RRIDs and transfection methodology.

Plasmids	Remarks	Plasmid transfection
pDRGFP (HR plasmid construct)	Gift from Maria Jasin<br>Addgene plasmid #26475; RRID:Addgene_26475	Cells were grown in 10 cm plates and transfection of pDRGFP (HR plasmid construct) and pimEJ5GFP (NHEJ plasmid construct) into HeLa cells were done by electroporation (Gene Pulser® Electroporation Systems, BIO-RAD). Prior to transfection, linearization of pimEJ5GFP was done. Quality control of the linearization was done by running gel electrophoresis in presence of non-linearized control plasmid. After electroporation, cells were grown on 20 cm plates with G418 or puromycin selection
pimEJ5GFP (NHEJ plasmid construct)	Gift from Jeremy Stark<br>Addgene plasmid # 44026 ; RRID:Addgene_44026	
pCBASceI	Gift from Maria Jasin<br>Addgene plasmid # 26477; RRID:Addgene_26477	

pcDNA3-EGFP	Gift from Doug Golenbock<br>Addgene plasmid #13031; RRID:Addgene_13031	for 7-10 days. Next, 15-20 colonies were isolated and grown in individual well on 6-wells plates. Selection of the most suitable colonies was performed by analyzing samples with flow cytometry.
mCherry2-C1	Gift from Michael Davidson<br>Addgene plasmid # 54563 ; RRID:Addgene_54563	
HPRT	Gift from James Thomson<br>Addgene plasmid #22884; RRID: Addgene_22884	

Supplementary Table S7. Gene set enrichment analysis of top upregulated (A) terms (IMX vs MX).

A.

Term	NES	Nominal p-value	FDR q-value
PRE-NOTCH EXPRESSION AND PROCESSING%REACTOME%R-HSA-1912422.1	2.28	0.0	0
NEGATIVE EPIGENETIC REGULATION OF RRNA EXPRESSION%REACTOME%R-HSA-5250941.2	2.24	0.0	0
DNA METHYLATION%REACTOME%R-HSA-5334118.1	2.22	0.0	0
POSITIVE EPIGENETIC REGULATION OF RRNA EXPRESSION%REACTOME%R-HSA-5250913.4	2.21	0.0	0
CONDENSATION OF PROPHASE CHROMOSOMES%REACTOME%R-HSA-2299718.1	2.21	0.0	0
CLEAVAGE OF THE DAMAGED PURINE%REACTOME%R-HSA-110331.2	2.17	0.0	0
SENESCENCE-ASSOCIATED SECRETORY PHENOTYPE (SASP)%REACTOME%R-HSA-2559582.2	2.13	0.0	9.44E-05
DEPURINATION%REACTOME%R-HSA-73927.1	2.12	0.0	8.86E-05
BASE-EXCISION REPAIR, AP SITE FORMATION%REACTOME DATABASE ID RELEASE 72%73929	2.07	0.0	1.31E-04
CLEAVAGE OF THE DAMAGED PYRIMIDINE%REACTOME DATABASE ID RELEASE 72%110329	2.06	0.0	1.75E-04
OXIDATIVE STRESS INDUCED SENESCENCE%REACTOME%R-HSA-2559580.4	2.05	0.0	2.92E-04
DEPYRIMIDINATION%REACTOME%R-HSA-73928.1	2.04	0.0	3.24E-04
CHROMATIN SILENCING%GOBP%GO:0006342	1.96	0.0	0.0015
GENE SILENCING BY RNA%REACTOME%R-HSA-211000.2	1.96	0.0	0.0015
NUCLEOSOME ASSEMBLY%REACTOME%R-HSA-774815.1	1.88	0.0	0.0087
DNA DAMAGE TELOMERE STRESS INDUCED SENESCENCE%REACTOME%R-HSA-2559586.3	1.83	0.0	0.0177
CHROMATIN MODIFYING ENZYMES%REACTOME%R-HSA-3247509.4	1.82	0.0	0.0237
BASE EXCISION REPAIR%REACTOME%R-HSA-73884.2	1.81	0.0017	0.0264
SIGNALING BY NOTCH%REACTOME DATABASE ID RELEASE 72%157118	1.78	0.0	0.0369
DNA DOUBLE STRAND BREAK RESPONSE%REACTOME DATABASE ID RELEASE 72%5693606	1.76	0.0	0.0509
HISTONE MODIFICATIONS%WIKIPATHWAYS_20200310%WP2369%HOMO SAPIENS	1.75	0.0	0.0540
REGULATION OF EPITHELIAL CELL APOPTOTIC PROCESS%GOBP%GO:1904035	1.74	0.0	0.0593

Supplementary Table S8. Gene set enrichment analysis of top downregulated (B) terms (IMX vs MX).

B.

Term	NES	Nominal p-value	FDR q-value
NUCLEOTIDE BIOSYNTHETIC PROCESS%GOBP%GO:0009165	-2.06	0.0	0.003
RIBONUCLEOTIDE BIOSYNTHETIC PROCESS%GOBP%GO:0009260	-2.06	0.0	0.002
G1 S TRANSITION%REACTOME DATABASE ID RELEASE 72%69206	-2.01	0.0	0.004
DNA REPLICATION%WIKIPATHWAYS_20200310%WP466%HOMO SAPIENS	-2.00	0.0	0.004
HALLMARK_G2M_CHECKPOINT%MSIGDB_C2%HALLMARK_G2M_CHECKPOINT	-2.00	0.0	0.004
RESOLUTION OF SISTER CHROMATID COHESION%REACTOME%R-HSA-2500257.1	-1.99	0.0	0.004
EML4 AND NUDC IN MITOTIC SPINDLE FORMATION%REACTOME DATABASE ID RELEASE 72%9648025	-1.98	0.0	0.005
MITOTIC SISTER CHROMATID SEGREGATION%GOBP%GO:0000070	-1.98	0.0	0.005
BARD1 SIGNALING EVENTS%PATHWAY INTERACTION DATABASE NCI-NATURE CURATED DATA%BARD1 SIGI	-1.96	0.0	0.006
SISTER CHROMATID SEGREGATION%GOBP%GO:0000819	-1.95	0.0	0.007
AMPLIFICATION OF SIGNAL FROM THE KINETOCHORES%REACTOME%R-HSA-141424.2	-1.94	0.0	0.007
MICROTUBULE CYTOSKELETON ORGANIZATION INVOLVED IN MITOSIS%GOBP%GO:1902850	-1.93	0.0	0.008
MITOTIC PROMETAPHASE%REACTOME DATABASE ID RELEASE 72%68877	-1.92	0.0	0.009
DNA REPLICATION%REACTOME%R-HSA-69306.4	-1.91	0.0	0.010
SEPARATION OF SISTER CHROMATIDS%REACTOME DATABASE ID RELEASE 72%2467813	-1.90	0.0	0.011
MITOTIC G1 PHASE AND G1 S TRANSITION%REACTOME DATABASE ID RELEASE 72%453279	-1.87	0.0	0.014
MITOTIC SPINDLE CHECKPOINT%REACTOME%R-HSA-69618.2	-1.86	0.0	0.015
HDR THROUGH HOMOLOGOUS RECOMBINATION (HRR)%REACTOME DATABASE ID RELEASE 72%5685942	-1.83	0.0023	0.021
BASE EXCISION REPAIR%WIKIPATHWAYS_20200310%WP4752%HOMO SAPIENS	-1.80	0.0	0.029
DNA-DEPENDENT DNA REPLICATION MAINTENANCE OF FIDELITY%GOBP%GO:0045005	-1.80	0.0	0.029
ERROR-PRONE TRANSLESION SYNTHESIS%GOBP%GO:0042276	-1.79	0.0069	0.031
INTERSTRAND CROSS-LINK REPAIR%GOBP%GO:0036297	-1.79	0.0021	0.032
RESOLUTION OF AP SITES VIA THE MULTIPLE-NUCLEOTIDE PATCH REPLACEMENT PATHWAY%REACTOME D/	-1.79	0.0	0.032
DOUBLE-STRAND BREAK REPAIR VIA HOMOLOGOUS RECOMBINATION%GOBP%GO:0000724	-1.78	0.0049	0.034
FANCONI ANEMIA PATHWAY%REACTOME%R-HSA-6783310.2	-1.78	0.0022	0.034
RESOLUTION OF D-LOOP STRUCTURES THROUGH SYNTHESIS-DEPENDENT STRAND ANNEALING (SDSA)%REA	-1.77	0.0021	0.035
BIOCARTA_ATRBRCA_PATHWAY%MSIGDB_C2%BIOCARTA_ATRBRCA_PATHWAY	-1.77	0.0	0.039
DNA MISMATCH REPAIR%WIKIPATHWAYS_20200310%WP531%HOMO SAPIENS	-1.74	0.0022	0.045
DNA DOUBLE-STRAND BREAK PROCESSING%GOBP%GO:0000729	-1.72	0.0067	0.053
REGULATION OF CELL CYCLE G2/M PHASE TRANSITION%GOBP%GO:1902749	-1.65	0.0	0.086
NEGATIVE REGULATION OF CELL CYCLE G2/M PHASE TRANSITION%GOBP%GO:1902750	-1.63	0.0	0.091
DNA IR-DOUBLE STRAND BREAKS (DSBS) AND CELLULAR RESPONSE VIA ATM%WIKIPATHWAYS_20200310%V	-1.63	0.0022	0.093
HDR THROUGH SINGLE STRAND ANNEALING (SSA)%REACTOME DATABASE ID RELEASE 72%5685938	-1.62	0.0	0.095
SPINDLE ORGANIZATION%GOBP%GO:0007051	-1.62	0.0	0.096
HDR THROUGH SINGLE STRAND ANNEALING (SSA)%REACTOME DATABASE ID RELEASE 72%5685938	-1.62	0.0	0.095
SPINDLE ORGANIZATION%GOBP%GO:0007051	-1.62	0.0	0.096

NES = Normalized enrichment score, FDR = False discovery rate. Nominal p-value of 0.0 is indicates an actual p-value of less than 1/number of 1000 permutations (i.e. < 0.001). Gene set databases are indicated in the term names.

Supplementary Figure S9.

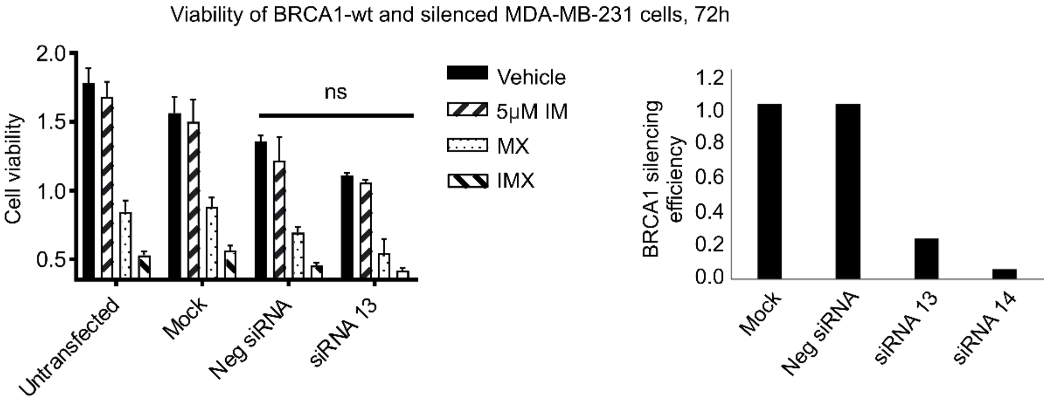

Supplementary Fig S9. IM reduces viability of MDA-MB-231 cells after DNA damage, regardless of BRCA1 status.

BRCA1 was silenced with siRNA. After 72 hours of treatment with vehicle, 5μM IM, 103.5nM MX and IMX (5μM IM + 103.5nM MX), cell viability was measured. The left bar chart shows cell viability under different treatments. The differences between neg-siRNA and siRNA13 were non-significant. The right graph shows silencing efficiency.

Supplementary Figure S10.

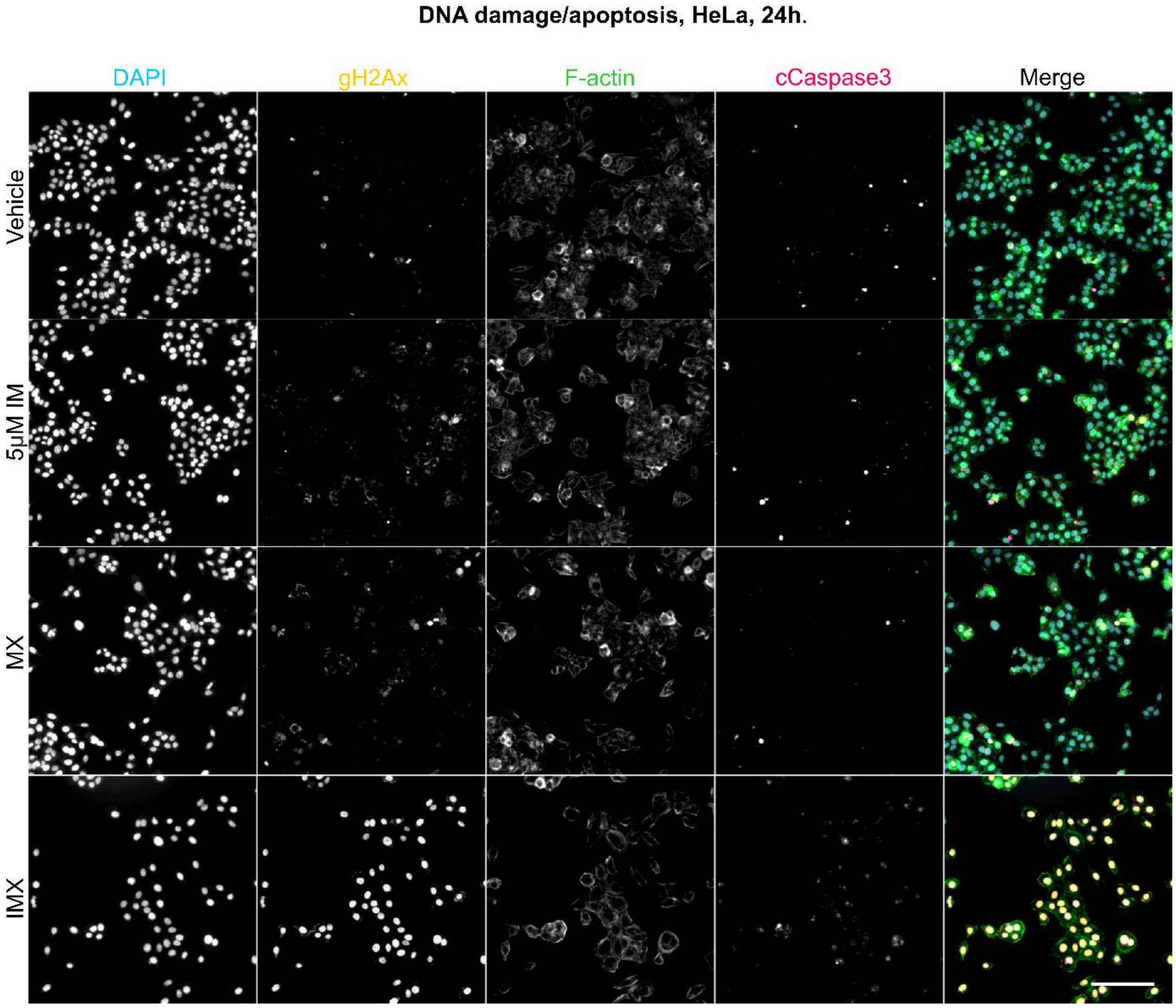

Supplementary Fig S10. IM increases DNA damage and apoptosis in cancer cells after MX treatment.

After 24 hours of treatment with vehicle, 5µM IM, 34nM MX and IMX (5µM IM + 34nM MX), HeLa cells were fixed and stained with DAPI, anti-γH2Ax (DNA damage marker) and anti-actin antibody for F-actin (cytoskeleton marker), anti-cleaved Caspase 3 antibody (apoptosis marker), followed by secondary antibody staining with anti-IgG-Alexa Fluor 568, anti-IgG-Alexa Fluor 488 and anti-IgG-Alexa Fluor 647. Bar represents 500 µm.

Supplementary Figure S11.

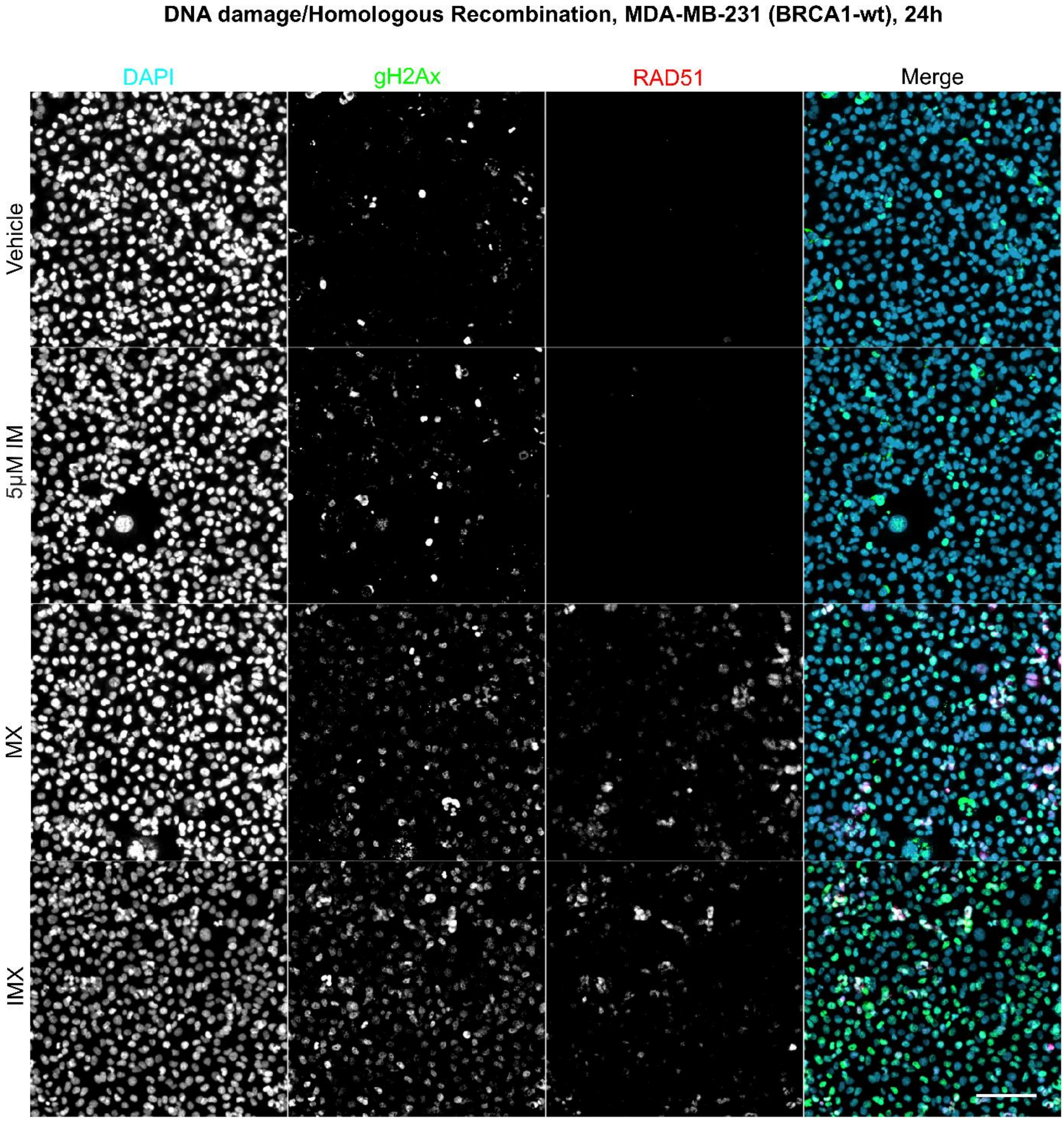

Supplementary Fig S11. IM increases DNA damage but reduces nuclear RAD51 levels in BRCA1-wt MDA-MB-231 cells.

After 24 hours of treatment with vehicle, 5µM IM, 103.5nM MX and IMX (5µM IM + 103.5nM MX), MDA-MB-231 (BRCA1-wt) cells were fixed and stained with DAPI, anti-γH2Ax and anti-RAD51 antibodies, followed by secondary staining with anti-IgG-Alexa Fluor 488 and anti-IgG-Alexa Fluor 647. γH2Ax and RAD51 are markers for DNA damage and homologous recombination, respectively. Bar represents 500 µm.

Supplementary Figure S12.

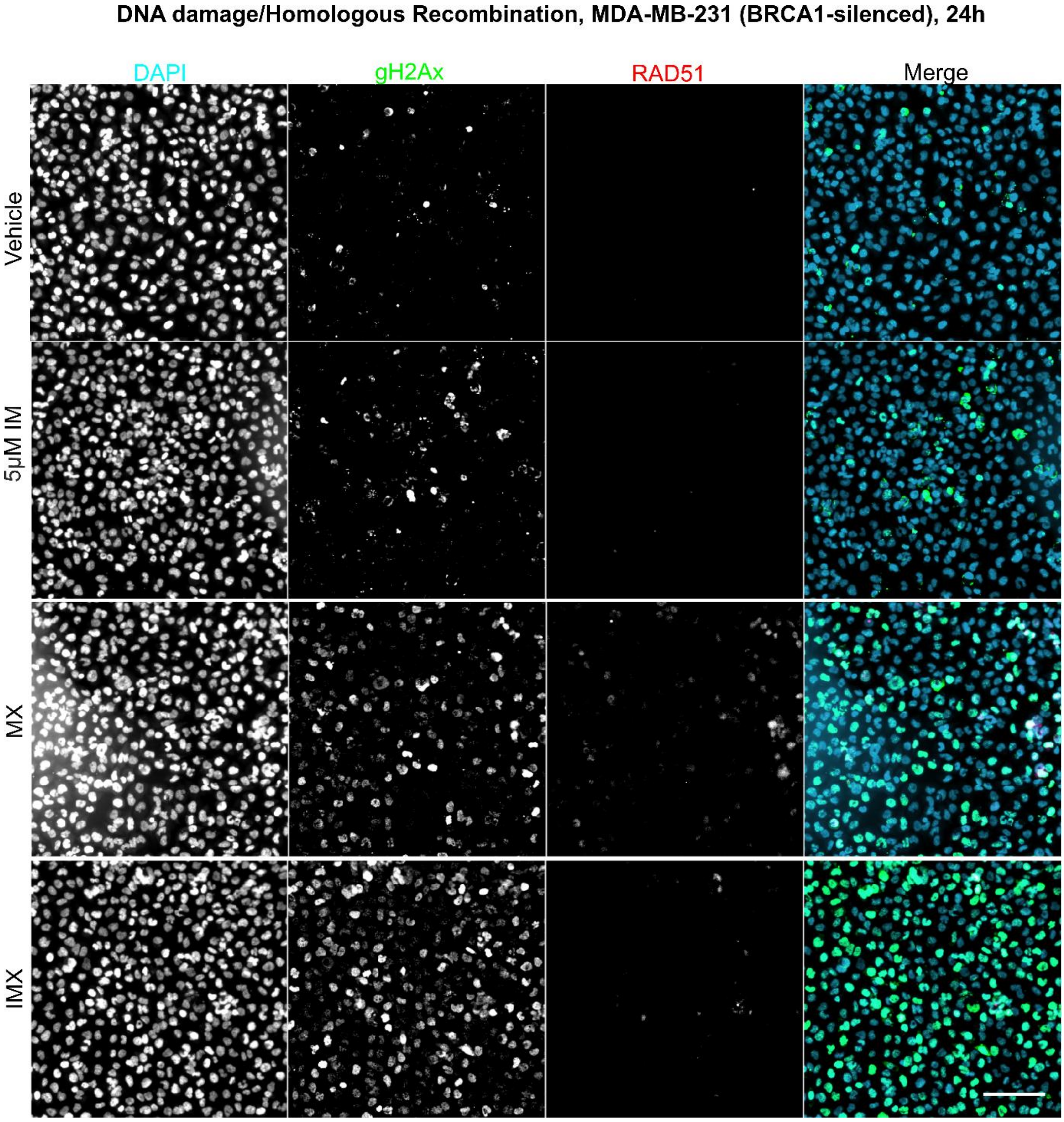

Supplementary Fig S12. IM increases DNA damage but reduces nuclear RAD51 levels in BRCA1-silenced MDA-MB-231 cells.

After 24 hours of treatment with vehicle, 5μM IM, 103.5nM MX and IMX (5μM IM + 103.5nM MX), BRCA1-silenced MDA-MB-231 cells were fixed and stained with DAPI, anti-γH2Ax and anti-RAD51 primary antibodies, followed by secondary antibody staining with donkey anti-IgG-Alexa Fluor 488 and anti-IgG-Alexa Fluor 647. γH2Ax and RAD51 are markers for DNA damage and homologous recombination, respectively. Bar represents 500 μm.

Supplementary Figure S13

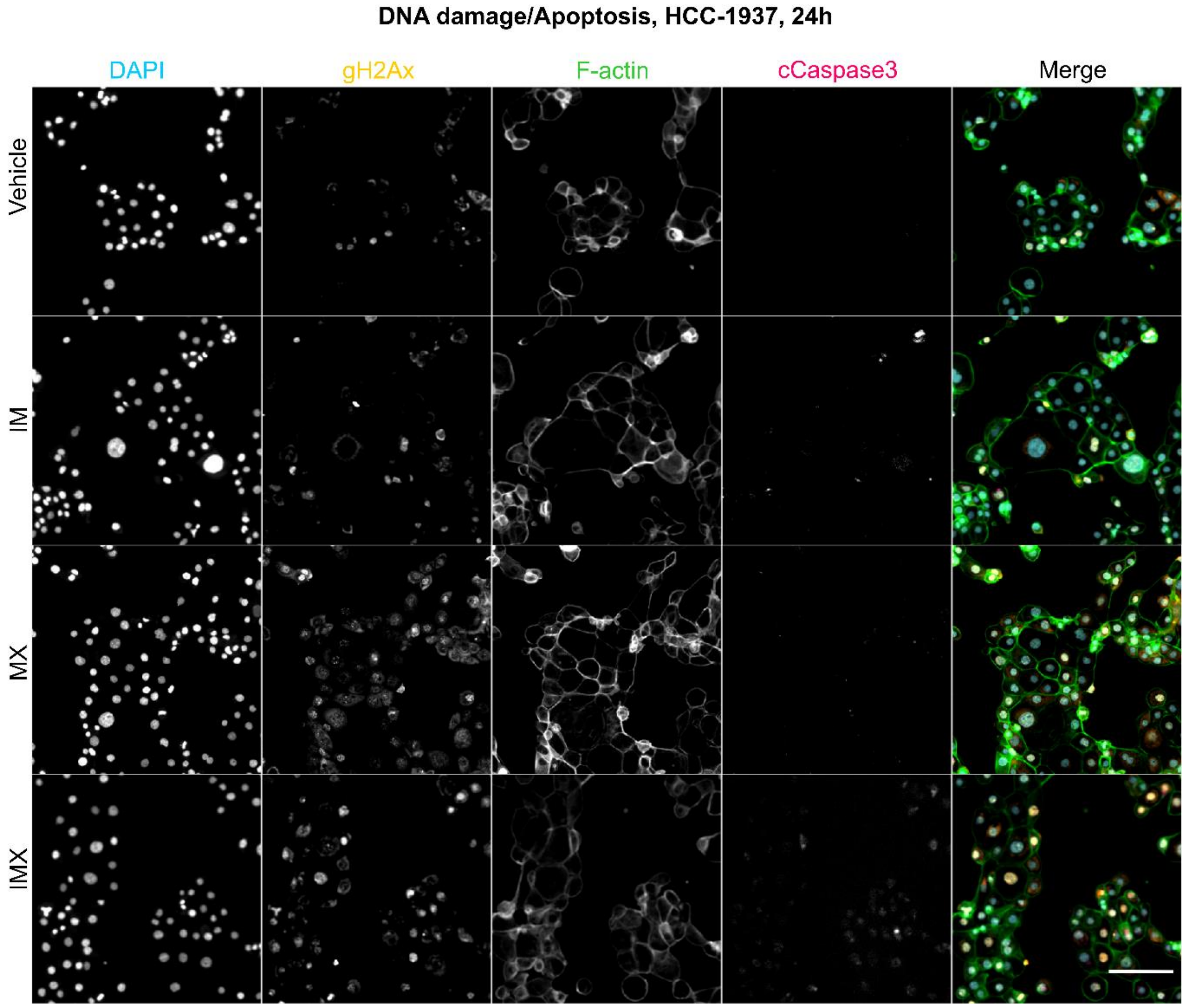

Supplementary Fig S13. IM increases DNA damage and apoptosis in BRCA1 mutant HCC-1937 cells after MX treatment.

After 24 hours of treatment with vehicle, 5µM IM, 9nM MX and IMX (5µM IM + 9nM MX), BRCA1 mutant HCC-1937 cells were fixed and stained with DAPI, anti-γH2Ax (DNA damage marker), anti-actin antibody for F-actin (cytoskeleton marker) and anti-cleaved Caspase-3 antibodies (apoptosis marker), followed by secondary antibody staining with anti-IgG-Alexa Fluor 568, anti- IgG-Alexa Fluor 488 and anti-IgG-Alexa Fluor 647. Bar represents 500 µm.

Supplementary Figure S14.

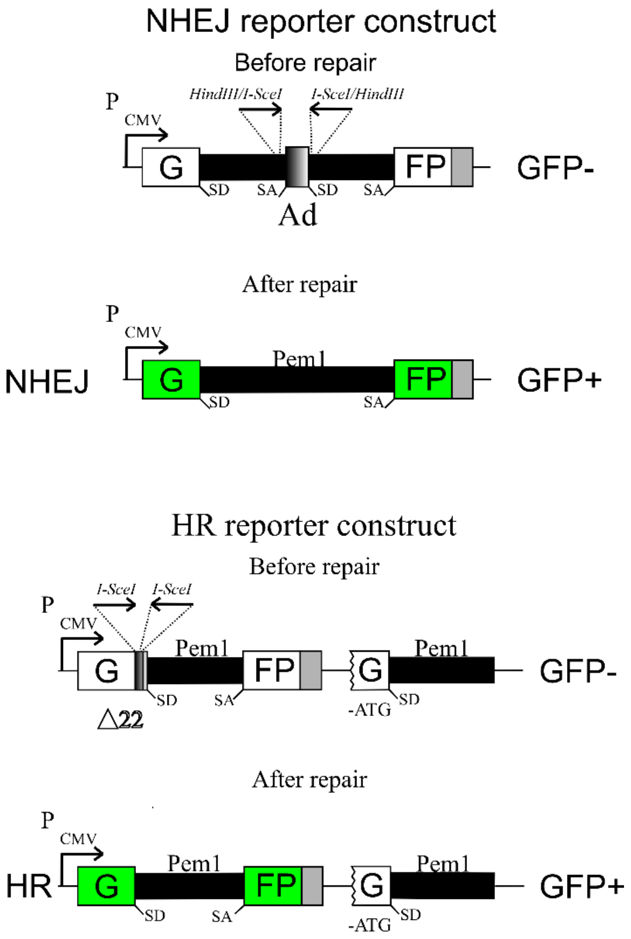

Supplementary Fig S14: Reporter constructs for the analysis of NHEJ and HR.

Upon DSB induction by I-SceI and successful NHEJ/HR, the constructs become GFP-positive. SD, splice donor; SA, splice acceptor.

Supplementary Figure S15

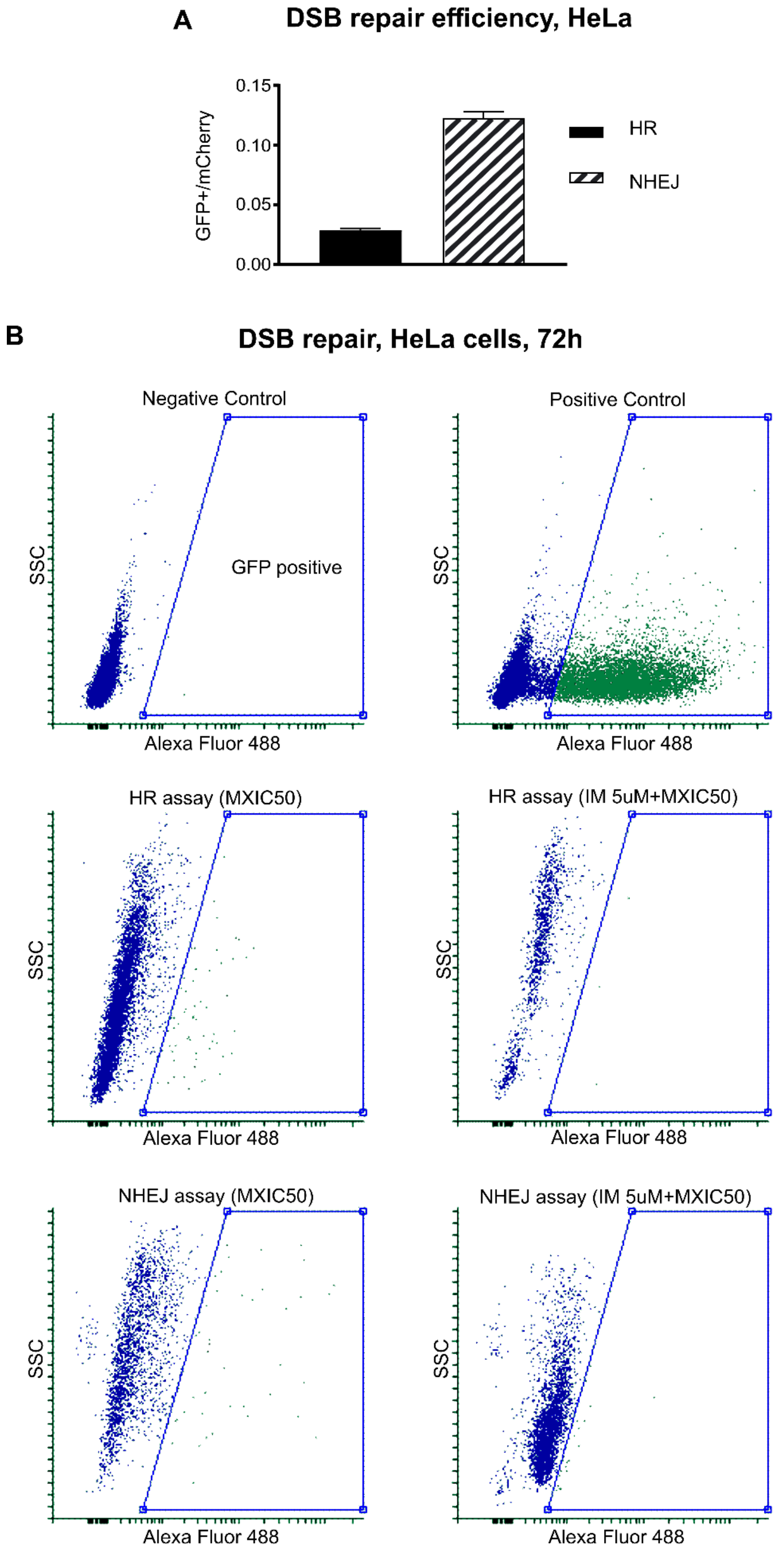

Supplementary Fig S15: DSB repair efficiency in HeLa cells.

A. NHEJ is more dominant repair process in HeLa cells. GFP signal is normalized against transfection efficiency control (mCherry).

B. Flow cytometry dot plots show Alexa Fluor 488 in X-axis and SSC in Y-axis. The gating for GFP+ cells is based on EGFP-positive control. The GFP+ cells are able to repair DNA damage via NHEJ or HR.

Supplementary Figure S16.

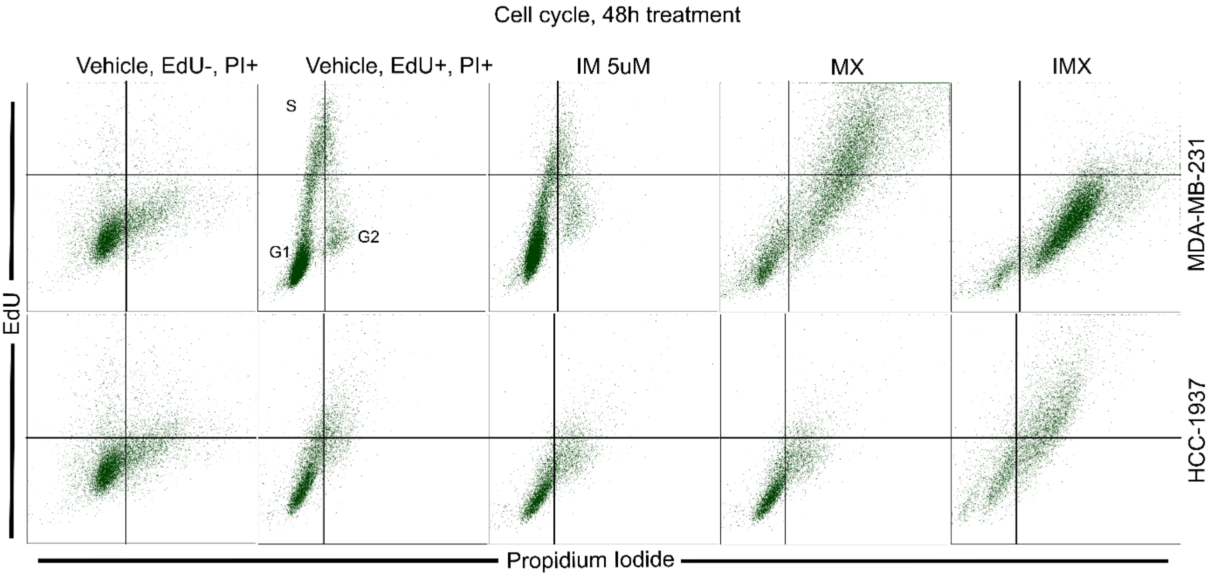

Supplementary Fig S16. IM causes G2 arrest in MDA-MB-231 and HCC-1937 cells after DNA damage.

After 48 hours of treatment with vehicle, 5µM IM, MX or IMX, cell pellets were collected and subjected to EdU-PI-based cell cycle analysis by flow cytometry. The dot plots show distribution of cell populations throughout the cell cycle for each cell line. Here, MX concentration for MDA-MB-231 is 103.5nM and for HCC-1937 is 9nM. IMX is the combination of 5µM IM and respective MX for each cell line.

Supplementary Video S17. DNA repair by HR and NHEJ in HeLa cells after MX treatment.

HeLa cells with HR and NHEJ reporter constructs were treated with 34nM MX and observed for 8 days. The video starts from 48h after the treatment. GFP+ cells were able to employ HR and NHEJ, and were therefore able go through mitosis and form colonies.

Supplementary Video S18. Cell cycle of FUCCI-HeLa cells.

FUCCI-HeLa cells were treated with vehicle, 5µM IM, 34nM MX and IMX (5µM IM + 34nM MX) for 72 hours. G1 cells have red fluorescence and S/G2/M cells have green fluorescence. Yellow cells are in G1/S transition.

Supplementary Table S19: IC_{50} of MX (with and without 5µM IM) in patient-derived HGSOC cell lines.

Cell line	Treatment condition	Tissue	BRCA status	SBS3 signature	HR status (Functional assay)	MX (nM) IC_{50}	95% CI	MX (nM) IC_{50} + 5µM IM	95% CI
M022p	primary	ascites	wt	Neg	HRP	645.5	458.6-901.8	165	143.5-190.1
M022i	interval	ascites	wt	Neg	HRP	861	562.9-1310	376	228.4-613.8
M048i	interval	omentum	wt	Neg	HRP	277	103.3-741.6	47	24.13-87.17
H002	interval	ascites	wt	Neg	HRP	1406	1184-1669	569.5	413.6-780.4
OC002	primary	ascites	wt	Neg	HRD*	523	353.5-755.6	146	122-174.7
interval = interval surgery after three courses of neoadjuvant treatment, primary = laparoscopy at diagnosis (no treatment), wt= wild type, HR= homologous recombination, HRP=HR-proficient, HRD=HR-deficient, *very low HRP score, MX=mitoxantrone, IM=imatinib, P<0.05, log (inhibitor) vs. normalized response-variable slope									

Supplementary Table S20: IC_{50} of MX (with and without 5µM IM) in conventional HGSOC cell lines.

Cell line	BRCA status	HR status (Functional assay)	Platinum sensitivity	MX (nM) IC_{50}	95% CI	MX (nM) IC_{50} + 5µM IM	95% CI
COV318	wt	HRP	Res	654	514.0-816.1	181	147.0-222.8
CaOV3	wt	HRP	Sens	118	89.83-154.3	29	24.40-33.95
OVCAR3	wt	HRP	Sens	329	254.0-423.8	96.5	76.32-120.9
OVCAR4	wt	HRP	Sens	270	194.1-372.1	65	42.81-96.46
OVCAR5	wt	HRP	Sens	284	174.0-457.8	56	36.88-83.52
COV362	splice donor mutation	HRD	Res	591	492.4-706.2	207	166-257.2
Kuramochi	BRCA2 nonsense mutation	HRD	Sens	695	555.3-863.1	195.5	151.4-250.8
OVCAR8	BRCA1 promoter methylation	HRD	Res	163	133.8-196.6	54	44.78-65.20
wt= wild type, HR= homologous recombination, HRP=HR-proficient, HRD=HR-deficient, MX=mitoxantrone, IM=imatinib, P<0.05, log (inhibitor) vs. normalized response-variable slope							
